# Bionic Microneedle Patch Inspired by Drosophila Tarsal Paws Boosts Healing in Bacterial Infectious Stomatitis

**DOI:** 10.1002/advs.202500432

**Published:** 2025-06-23

**Authors:** Jiaqi Qin, Cewen Hu, Xiaohong Ran, Huajing Zeng, Jie Zhang, Bin Liu, Zengjie Fan

**Affiliations:** ^1^ Key Laboratory of Dental Maxillofacial Reconstruction and Biological Intelligence Manufacturing School of Stomatology Lanzhou University Lanzhou Gansu 730000 P. R. China

**Keywords:** antibacterial, anti‐inflammatory, bacterial infectious stomatitis, bionic microneedle patch, suction cup hydrogel

## Abstract

Bacterial infectious stomatitis (BIS), characterized by severe infections overlaying ulcers, poses complex therapeutic challenges and often results in prolonged healing, leading to significant patient discomfort. To address this clinical challenge, a biomimetic microneedle (MN) suction cup is designed inspired by the tarsal claw of Drosophila. The suction cup component is crafted from polyvinyl alcohol (PVA) hydrogel, while the MN section, embedded within the suction cup, comprises a thermoresponsive and antibacterial composite hydrogel infused with the drug S‐nitrosoglutathione (GSNO). This innovative design not only ensures strong adhesion to moist surfaces but also provides substantial antibacterial and anti‐inflammatory effects, controlled drug release, active promotion of concentric wound contraction, and enhanced vasodilation, thereby facilitating rapid and comprehensive healing of BIS. The multifunctionality of the MN suction cup patch developed in this study represents a significant innovation over traditional treatment methods and holds potential for widespread application in dental clinics.

## Graphical abstract

Microneedle suction cup patches, prepared using bionic Drosophila tarsal claws, can effectively treat bacterial infectious stomatitis by rapidly and efficiently leveraging multiple effects, including antibacterial and anti‐inflammatory properties, promotion of vasodilation, and active mechanical contraction of the wound.

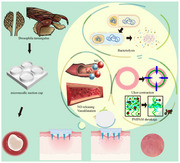

## 1 Introduction

Bacterial infectious stomatitis (BIS) is an acute bacterial condition caused by pathogens such as *S. aureus*, *S. viridans*, and *E. coli*. It is pathologically characterized by dense pseudomembranes overlaying ulcerative lesions, composed primarily of necrotic tissue, inflammatory cells, and fibrin.^[^ ^1^ ^]^ This condition disproportionately affects vulnerable populations, particularly neonates and the elderly. Clinically, BIS manifests as fever, oral pain, sore throat, drooling, and the formation of oral pseudomembranes.^[^ ^1^ ^]^ Current therapeutic strategies, including pharmaceutical treatments such as chlorhexidine rinses, systemic and topical glucocorticoids, growth factors, and analgesics, as well as advanced modalities like laser therapy, frequently fail to expedite ulcer healing.^[^ ^2^, ^3^, ^4^ ^]^ Consequently, these treatments often require complex and costly multifaceted regimens.

A promising avenue to enhance treatment efficacy involves the application of hydrogels. These hydrophilic polymers can incorporate bioactive functionalities, including anti‐inflammatory and antimicrobial agents, to accelerate ulcer healing.^[^ ^5^, ^6^ ^]^ However, the wet environment of the oral cavity presents challenges for hydrogel application, particularly concerning their solubility and adhesive properties.^[^ ^7^ ^]^ Innovations in biomimicry offer novel strategies to improve hydrogel adhesion in moist conditions. Inspired by natural adhesion mechanisms, such as the unique tarsal claw architecture of Drosophila melanogaster, scientists are developing innovative design concepts. These claws generate negative pressure through dual flat suction cup, with tarsal hairs enhancing adherence through micro‐interlocking.^[^ ^8^, ^9^ ^]^ By leveraging these biological principles, integrating hydrogels with micro‐ and nanofabrication technologies can significantly augment their efficacy. For instance, MN technology provides minimally invasive, pain‐free, and targeted drug delivery.^[^ ^10^, ^11^ ^]^ However, achieving stable adhesion in the oral cavity remains challenging, as conventional MN patches rely primarily on chemical bonding, susceptible to mechanical detachment. The combination of suction cup technology with MN offers a substantial advancement, forming a composite structure with robust wet adhesion and targeted drug delivery.

Selecting appropriate materials for the suction cup is crucial. Polyvinyl alcohol (PVA) is particularly well‐suited for this purpose due to its strong adhesion, biocompatibility, and cost‐effectiveness, making it ideal for fabricating hydrogel MN suction cup patches. PVA forms effective chemical bonds with the oral mucosa, ensuring stable attachment in the humid oral environment.^[^ ^12^ ^]^ For the MN's hydrogel component, materials with excellent mechanical properties are essential to ensure skin penetration. Carboxymethyl cellulose sodium (CMC‐Na), a cellulose derivative, exhibits outstanding biodegradability and mechanical strength when incorporated into hydrogel formulations. The integration of CMC‐Na strategically enhances the hydrogel's mechanical properties, enabling the MN to withstand the demands of penetrating the oral mucosa.^[^ ^13^ ^]^

In addition to mechanical resilience, the MN should possess significant antibacterial properties to inhibit bacterial growth around the ulcer. Hydroxypropyltrimethyl ammonium chloride chitosan (HACC), a chitosan derivative, displays notable antibacterial activity while maintaining biocompatibility, biodegradability, and hemostatic properties, all of which are vital for promoting wound healing.^[^ ^14^, ^15^ ^]^ HACC's quaternary ammonium groups act as surface‐active cations, interacting with biological surfaces to alter membrane permeability, denature proteins and enzymes, and disrupt cellular metabolism, leading to microbial autolysis.^[^ ^16^ ^]^

Furthermore, anti‐inflammatory and pro‐regenerative properties are essential. As an endogenous nitric oxide (NO) donor, S‐nitrosoglutathione (GSNO) demonstrates high biocompatibility and low systemic toxicity. Its inherent stability under physiological conditions allows for a gradual release of NO, effectively avoiding issues associated with rapid degradation and toxic NO accumulation. GSNO also achieves cellular targeting through glutathione transporters (e.g., MRP1), selectively accumulating in endothelial and immune cells for spatially controlled NO delivery. Furthermore, GSNO demonstrates intrinsic antioxidant activity by scavenging reactive oxygen species (ROS) and mitigating oxidative stress damage. These synergistic properties—sustained release kinetics, cell‐type specificity, and dual functionality in redox modulation—collectively support the selection of GSNO as the optimal NO donor in our experimental design.^[^ ^17^, ^18^, ^19^ ^]^ The NO released from GSNO acts as a signaling molecule that interacts with the vascular endothelium to induce vasodilation and regulate inflammatory responses. It activates protein kinase G, leading to a decrease in calcium ion concentration, which promotes relaxation of vascular smooth muscle and triggers vasodilation. Consequently, the dilated blood vessels enhance tissue blood flow and improve the supply of oxygen and nutrients. Additionally, NO can inhibit the NF‐κB pathway, reducing the production of pro‐inflammatory cytokines (e.g., tumor necrosis factor‐alpha [TNF‐α] and interleukin‐6 [IL‐6]) and decreasing the migration of leukocytes to the inflammation site. NO plays a crucial role in regulating the immune response by enhancing the phagocytosis and bactericidal functions of macrophages, inhibiting T‐cell proliferation and differentiation, and controlling infection and inflammation, thereby promoting ulcer repair.^[^ ^17^, ^20^, ^21^ ^]^

To enhance the bioavailability of GSNO, effectively controlling its release is essential.^[^ ^22^, ^23^ ^]^ Smart responsive materials, particularly those that respond to temperature, provide advanced solutions for controlled drug delivery. Among these materials, poly(N‐isopropylacrylamide) (PNIPAM)‐based hydrogels exhibit distinct advantages over other temperature‐responsive materials such as poly(ethylene glycol) (PEG)‐based copolymers and poloxamers (Pluronics).^[^ ^24^, ^25^ ^]^ PNIPAM stands out due to its precise and predictable phase transition at ≈32 °C, close to human body temperature. This allows PNIPAM to efficiently control the release of encapsulated drugs.^[^ ^24^ ^]^ Upon reaching its critical transition temperature, PNIPAM undergoes a significant transformation from a coiled to a globular state, enabling a rapid initial release of GSNO. This burst release is followed by a sustained, locally diffused release over time, ensuring a high concentration of the drug at the site of application and enhancing therapeutic efficacy.^[^ ^25^ ^]^ This dual‐phase release mechanism offers a distinct advantage over other thermoresponsive materials which mostly rely on gradual diffusion. In addition to controlled drug release, PNIPAM also plays a crucial role in promoting wound healing through enhanced wound contraction. The unique phase transition of PNIPAM induces contractile forces that facilitate a purse‐string‐like approximation of wound margins, thereby promoting tissue closure and reducing healing time.^[^ ^18^ ^]^ These combined properties make PNIPAM not only an effective agent for controlled drug release but also a valuable material in accelerating wound healing and contraction.

In this study, we aimed to develop a bioinspired hydrogel MN suction cup patch that synergistically integrates the strong wet adhesive capabilities of a suction cup with the precision of MNs for targeted drug delivery. This innovative design leverages HACC to provide potent antimicrobial properties, while thermoresponsive PNIPAM is utilized to regulate the controlled release of NO and accelerate the concentric contraction of ulcer wounds, thereby enhancing the body's natural ulcer healing processes. The biomimetic hydrogel MN suction cup exhibited excellent biocompatibility and significant antimicrobial activity, promoting accelerated tissue repair in ulcerated regions. These features underscore its potential as an effective therapeutic approach for treating complex oral ulcerations, especially those complicated by infectious conditions such as BIS.

## 2 Results

### 2.1 Fabrication and Function of MN Suction Cup

**Scheme 1:**
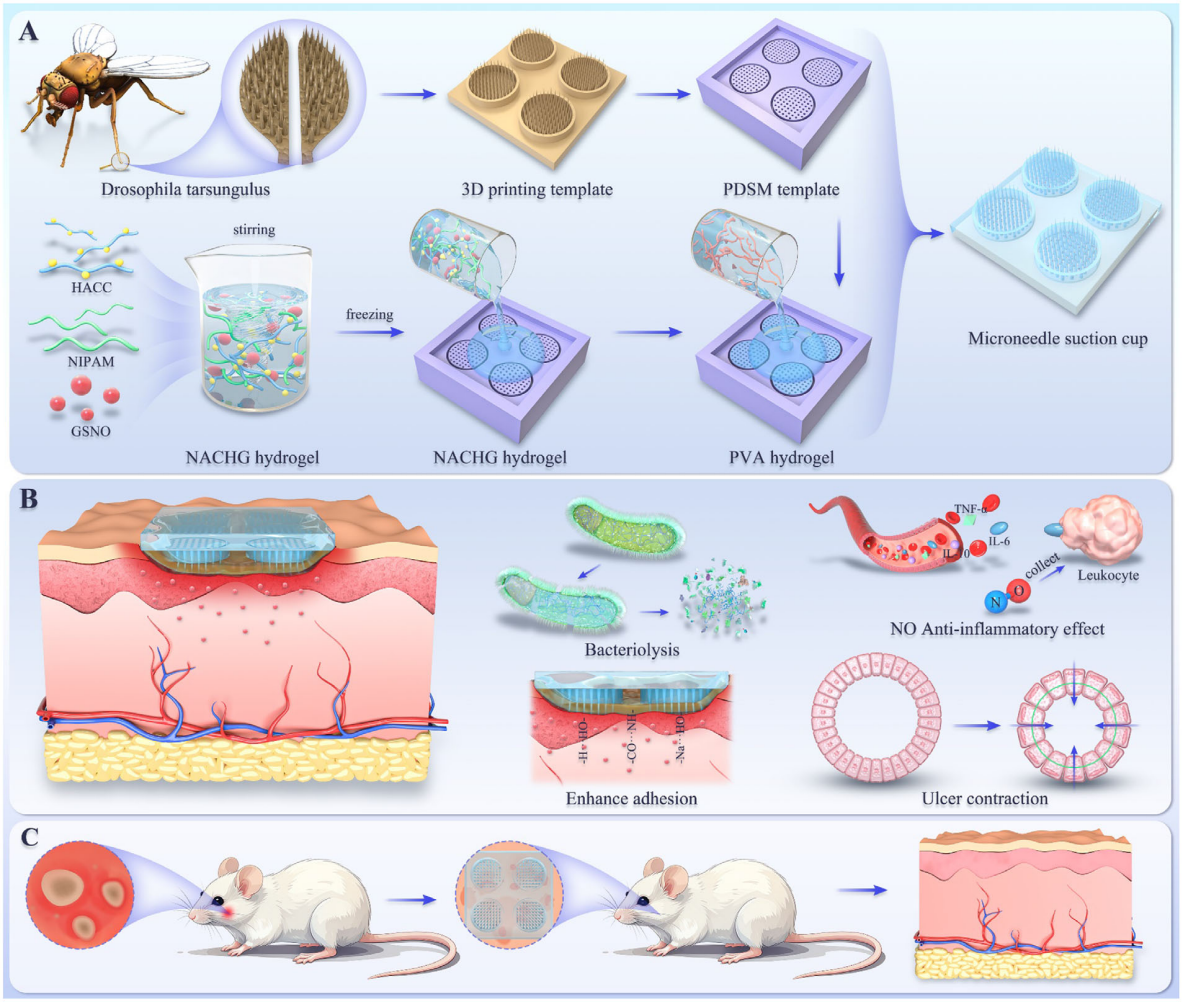
Schematic diagram of MN suction cup. A) Schematic diagram of microneedle suction cup synthesis. B) Microneedle suction cup adhesion and functioning principle. C) Ulcer healing process in the rat BIS model.

**Figure 1:**
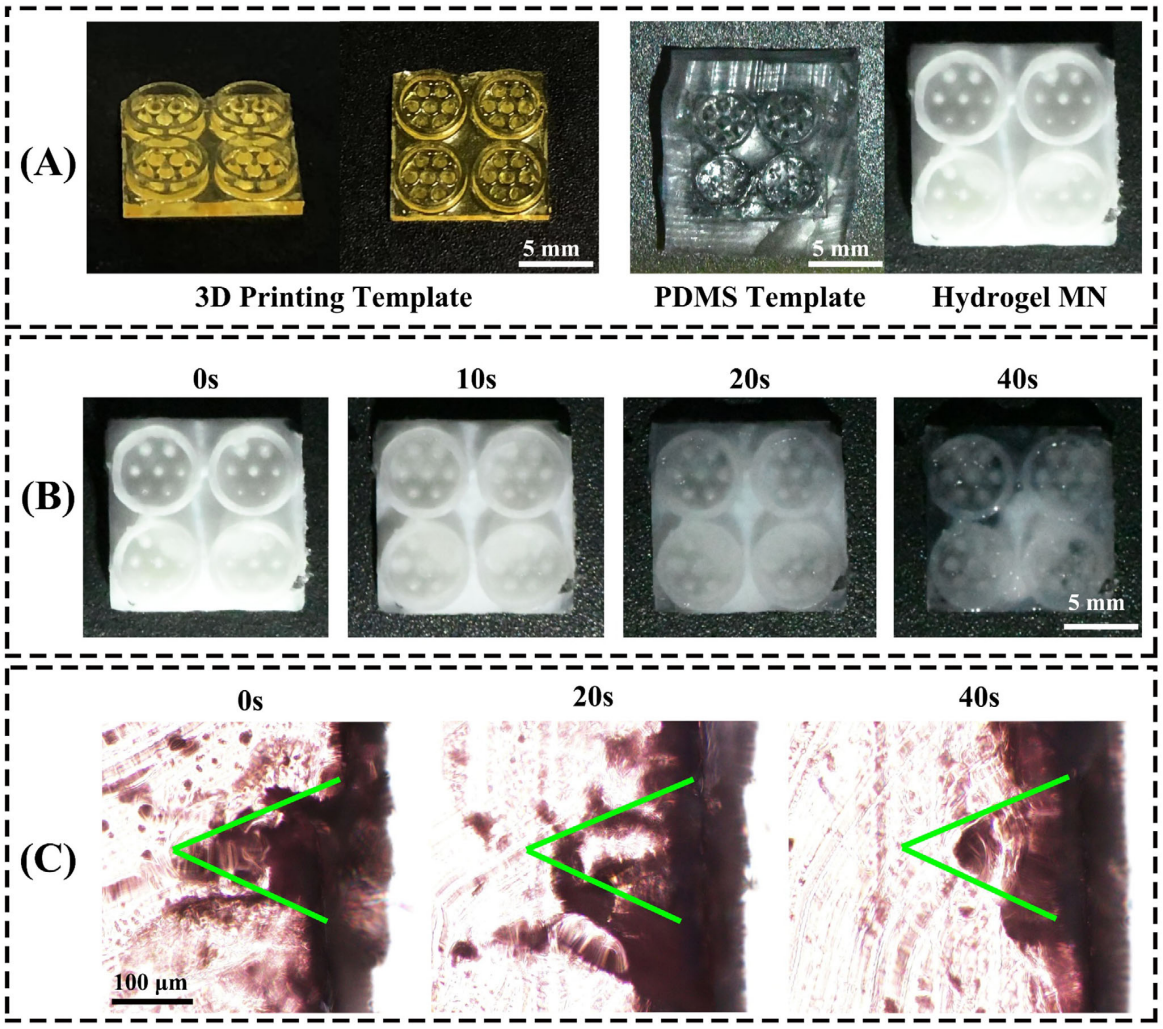
Characterizations of MN suction cup. A) The resin and PDMS models, and hydrogel samples of MN suction cup (Scale bar = 5 mm). B) MN suction cup thawing process, taken as 0, 10, 20, and 40 s (Scale bar = 5 mm). C) Gelatin puncture ability, taken as 0, 20, and 40 s (Scale bar = 100 µm).

As illustrated in **Scheme** **1**, inspired by the proficient adhesion of Drosophila's tarsal claw to smooth surfaces, we integrated MN and biomimetic suction cup technologies to construct a MN suction cup patch using a resin template prepared through 3D micro‐nano printing technology (MicroArch S230). This integration aims to overcome the low moisture‐adhesive ability of hydrogels while providing a minimally invasive and painless delivery method. Based on the resin template, a polydimethylsiloxane (PDMS) negative replica was fabricated (**Figure** **1**A). Subsequently, the thermoresponsive and antibacterial composite hydrogel loaded with GSNO (abbreviated as NACHG hydrogel) was infused into the MN component. After the NACHG hydrogel solidified under cooled conditions, PVA hydrogel was introduced to form the suction cup segment (Scheme 1A). The prepared MN suction cup patch was then utilized to treat the BIS in a rat model. The enhanced tissue adhesion provided by the biomimetic MN, the synergistic effects of NO's anti‐inflammatory action, controlled drug release from the hydrogel, and the hydrogel's concentric contraction all contributed to accelerating the healing of BIS (Scheme 1B,C).

A systematic experimental design comprised six distinct groups, A CMC‐Na‐enhanced cross‐linked network of PNIPAM in combination with PAA was used, with the mass of HACC added at 50, 100, and 150 mg, and incorporating GSNO at two concentrations (100 and 500 µm) within each ratio. These groups were designated as NACH1G0.04, NACH1G0.2, NACH2G0.04, NACH2G0.2, NACH3G0.04, and NACH3G0.2 (Table S1, Supporting Information). Upon thawing at ambient temperature after 40 s, the frozen MN suction cup began to show significant melting at 20 s (Figure 1B). The MN suction cups had good adhesion properties, notably, even when bent beyond 90° to 135°, the structural integrity was maintained, evidencing remarkable flexibility and bond strength (Figure S1A, Supporting Information). In vitro puncture tests were conducted on gelatin, which was stained for enhanced visualization and whose mechanical properties approximated those of oral mucosa. These assays aimed to evaluate the penetrative capacity of the fabricated MN. Within the initial 20 s of application, the MN demonstrated substantial piercing efficacy. This observation was further corroborated by analysis of gelatin sections, which showed that the intact morphology of the MN was visibly preserved along their entire length in the longitudinal view (Figure 1C), highlighting the successful retention of MN architecture post‐puncture.

### 2.2 Morphological and Composition Characterization of MN Suction Cup

The PVA suction cup, derived through PVA freeze‐thaw hydrogen bond cross‐linking, exhibit favorable adhesiveness in wet environments. This adhesiveness is primarily attributed to two mechanisms: i) chemisorption‐induced bond formation and ii) the utilization of atmospheric pressure principles. Post‐polymerization, PVA manifests an abundance of hydroxyl groups (‐OH), which facilitate extensive hydrogen bonding with the aqueous molecules present within the oral mucosal mucus layer, thereby enhancing adhesive characteristics.^[^ ^26^, ^27^ ^]^ Moreover, these hydroxyl groups are prone to engage in covalent bond formation with various oral cavity constituents, including carboxyl and isocyanate groups, thereby establishing robust, enduring connections. Furthermore, the evacuation of air from the PVA suction cup engenders a negative pressure environment internally. This strategic design leverages atmospheric pressure to significantly amplify the adhesive performance of the cup under wet conditions, ensuring steadfast adherence even in the challenging oral environment. Upon air evacuation, the MN component of the suction cup achieves subtle penetration into the mucosal layer, while concurrently, the hydrogel constituent of the MN region contributes to adhesive efficacy under damp conditions.

**Figure 2:**
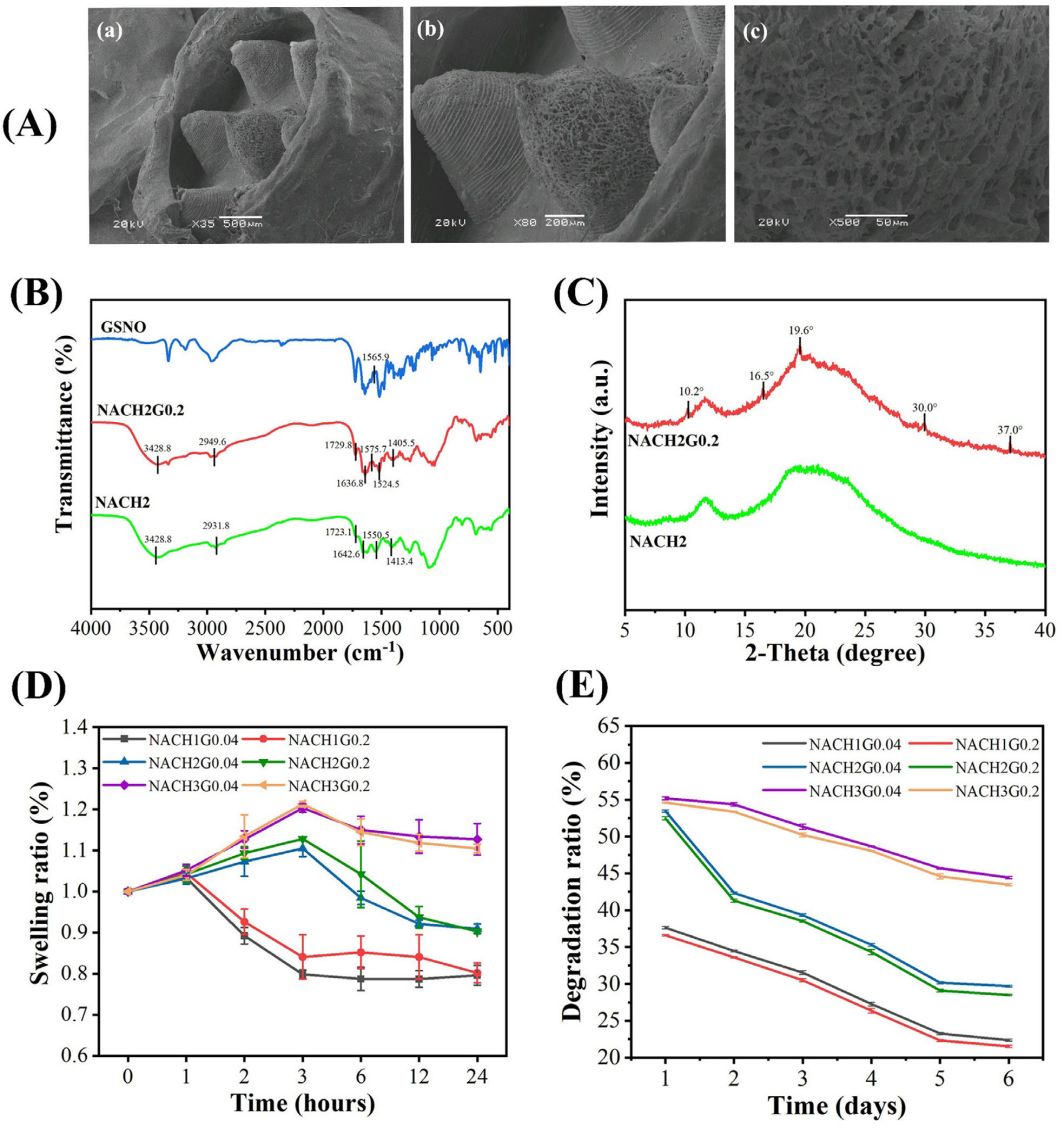
Characterizations of morphology and mechanical properties of MN suction cup. A) SEM images of MN suction cup patch (scale bars = 500, 200, and 50 µm). B) FTIR of MN hydrogel. C) XRD spectrum of MN hydrogel. D) Swelling rate of MN hydrogels in 6 groups with different proportions within 24 h. (*n* = 3). E) Degradation rates of 6 groups with different proportions of MN hydrogels in 6 days. (*n* = 3).

Scanning electron microscopy (SEM) inspections reveal a meticulously formed MN architecture situated centrally within the suction cup, affirming the completion of hydrogel cross‐linking processes (**Figure** **2**A). Fourier Transform Infrared (FTIR) spectroscopic analysis further attests to the establishment of a NACHG hydrogel cross‐linked matrix, evidenced by the presence of characteristic absorption bands indicative of amide group interactions: 1524.5 cm⁻¹ for N─H stretching, 1636.8 cm⁻¹ for C═O stretching, 1405.5 cm⁻¹ for C─N stretching vibrations, alongside 2949.6 and 3428.8 cm⁻¹ bands suggestive of N‐H bending and O─H bonds, respectively. And the ‐COOH characteristic peak is at 1729.8 cm^−1^. These findings confirm the constitution of the hydrogel's cross‐linked framework (Figure 2B). Notably, the detection of N═O stretching vibrations ≈1600 cm⁻¹ signifies the dispersion of GSNO within the hydrogel matrix and its potential for NO release.^[^ ^28^ ^]^ The NACHG hydrogel has an amorphous structure, and multiple diffraction peaks appeared on the spectrum of the hydrogel after compounding with GSNO.^[^ ^29^ ^]^ X‐ray diffraction (XRD ）) patterns exhibit distinct peaks at 10.2, 16.5, and 19.6°, affirming the successful loading of GSNO into the hydrogel network (Figure 2C). Post MN penetration into the mucosa, H‐bonds, and amide linkages within the NACHG hydrogel facilitate adhesion via chemical bonding.^[^ ^30^ ^]^ The synergistic action of adhesive mechanisms in the suction cup segment (including atmospheric pressure effects), coupled with the chemical bonding facilitated by the MN component, significantly bolsters the adhesive performance of the suction cup MN construct. Consequently, this design ensures robust bonding capacity even in the moist environment of the oral mucosa (Scheme 1B).

### 2.3 Swelling, Degradation, Shrinkage, and Mechanical Characterizations of MN Hydrogels

Different groups were created by varying the compositional ratios of PNIPAM and HACC, resulting in the formation of six distinct formulations, from which the optimal ratio was selected for further experimentation. These six hydrogel formulations, with varying compositional ratios, were evaluated for their swelling characteristics in artificial saliva. Notably, a positive correlation was observed between the proportion of HACC and the swelling rate over a 24‐h period, with higher concentrations yielding increased swelling. Conversely, the group with the lowest HACC content (NACH1G0.04 and NACH1G0.2) exhibited ≈10% dissolution after 2 h, stabilizing at an 80% swelling ratio by 24 h. The moderately HACC group (NACH2G0.04 and NACH2G0.2) began dissolving within the initial 3 h, subsequently proceeding at a gradual pace, reaching a 10% reduction after 24 h (Figure 2D). In the degradation study, following a single day of incubation, both the highest HACC content and moderately HACC content ratio groups exhibited approximately 45% degradation, whereas the lowest HACC content ratio group demonstrated more substantial degradation of 62.5%. The degradation rate of the moderately HACC content ratio group intensified over time, ultimately resulting in substantial degradation, whereas ≈45% of the highest HACC content ratio group remained intact even after 6 days. The lowest HACC content ratio group underwent nearly complete degradation, albeit with an initial phase characterized by excessively rapid disintegration (Figure 2E). Consequently, the lowest HACC content ratio group is not considered an ideal candidate for the optimal formulation. Chitosan, a hydrophilic polymer, contributes to the network architecture of the hydrogel, with its aggregation in aqueous media facilitated by hydrophilic interactions, thereby promoting the swelling of the hydrogel upon water absorption.^[^ ^31^ ^]^ However, this inherent property imparts a resistance to degradation in moist environments such as the oral cavity. Hence, when the chitosan content within the hydrogel matrix is diminished, an accelerated degradation rate becomes a prominent issue. Ideally, oral hydrogels should exhibit a balanced swelling capacity without excessive expansion and maintain a controlled degradation profile to ensure prolonged functionality. Considering these criteria, the moderately HACC content ratio group emerges as a more suitable formulation, striking a favorable balance between swelling moderation and degradation kinetics conducive to oral applications.

**Figure 3:**
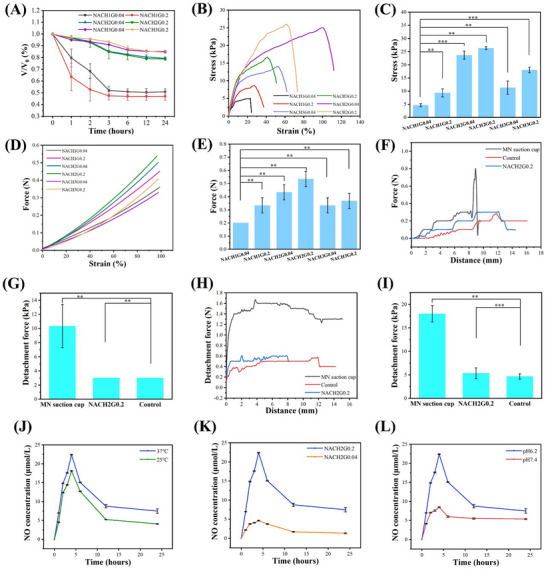
Mechanical characterization and drug release assay of MN suction cup. A) Proportion of thermal shrinkage for six groups of MN hydrogels with different compositions over 24 h at 37 °C. B) Compression properties of MN across six groups with varying ratios. C) Maximum values of MN compression properties for the six groups with different ratios. D) Tensile properties of MN hydrogels across six groups with varying ratios. E) Maximum values of MN tensile properties for the six groups with different ratios. F) Shear properties of MN suction cup, NACH2G0.2, and control groups after bonding to rat oral mucosa. G) Maximum values of shear properties for MN suction cup, NACH2G0.2, and control groups after bonding to rat oral mucosa. H) Peeling properties of MN suction cup, NACH2G0.2, and control groups after bonding to rat oral mucosa. I) Maximum values of peeling properties for MN suction cup, NACH2G0.2, and control groups after bonding to rat oral mucosa. J) NO release at 37 and 25 °C. K) NO release from NACH2G0.2 and NACH2G0.04. L) NO release at pH 6.2 and 7.4, respectively (*n* = 3; **P < 0.05; ***P < 0.001).

The hydrogel is engineered to facilitate the active release of GSNO at physiological temperature (37 °C), a function enabled by the incorporation of PNIPAM, thereby capitalizing on its thermoresponsive characteristics.^[^ ^32^ ^]^ This endows the hydrogel with the capability to exert a contraction force upon the ulcer's peripheral tissue, theoretically augmenting the healing process through the application of mechanical stimuli. In contrast to the substantial contraction exhibited by PNIPAM at 37 °C, PNIPAM showed no significant contraction characteristics over 24 h at 25 °C. This suggests that the NACHG hydrogel shrinks to release the drug on contact with an inflamed wound, while maintaining the drug in the hydrogel at room temperature. Notably, the pronounced contraction observed in the lowest HACC content composition is attributed to an undesirably rapid degradation rate, rendering this formulation unsuitable for selection. Conversely, the moderately HACC content and highest HACC content formulations exhibit contraction profiles that are remarkably similar, displaying shrinkage percentages of 21.2% and 15.7%, respectively, indicative of consistent contraction dynamics (Figure S2A, Supporting Information). Both formulations follow a temporal trend characterized by an initial increase in shrinkage that plateaus ≈12 h post‐application. Of particular interest, the moderately HACC content formulation achieves a contraction rate of 15.3% within a mere 3 h timeframe, fulfilling the therapeutic requirements effectively (**Figure** **3**A). This underscores the moderately HACC content composition's suitability for the intended therapeutic intervention given its expedited yet sustained contraction behavior.

The compressive strength assessment of the MN was conducted following partial cryo‐solidification, with the evaluation centered on the MN facet. At a strain magnitude of 100%, the moderately HACC content formulation attained a peak stress value of 0.53 N, highlighting its mechanical robustness. Given the documented load‐bearing capacity range of oral mucosa, which spans from 0.3 to 0.37 N, the peak penetration capability of the moderately HACC content formulation is deemed adequate for the intended therapeutic intervention (Figure 3B,C). The observed superiority of the moderately HACC content composition over the lowest HACC content and the highest HACC content counterparts is intricately linked to the optimized balance of HACC content and CMC‐Na. A lower HACC proportion results in a hydrogel matrix that is insufficiently compact, exhibiting heightened porosity and compromised structural integrity (Figure S2B,C, Supporting Information). With escalating concentrations of chitosan, an increase in interchain hydrogen bonding among the side‐chain hydroxyl groups ensues, giving rise to a denser and more intricately entangled matrix.^[^ ^15^, ^16^ ^]^ Consequently, at diminished chitosan concentrations, a decline in the number of cohesional and entanglement sites occurs, culminating in the formation of a relatively looser structural arrangement.^[^ ^15^, ^16^ ^]^ Conversely, an excess of HACC diminishes the interplay between PNIPAM and HACC, leading to excessive intra‐HACC crosslinking that degrades the overall hydrogel strength. Incorporation of CMC‐Na within the moderately HACC content formulation group exhibited a substantial augmentation in compressive strength relative to formulations devoid of CMC‐Na. Specifically, the peak compressive force recorded in the absence of CMC‐Na was observed to be 0.2 N. Upon introduction of CMC‐Na, this peak compressive strength experienced a 2.5‐fold increase, thereby achieving a magnitude conducive to facilitating penetration through the oral mucosal barrier, thus aligning with the design prerequisites for effective buccal delivery systems (Figure S2D,E, Supporting Information). The integration of CMC‐Na serves a dual purpose: it mitigates water volatility, thereby fostering an environment conducive to enhanced intermolecular interactions.^[^ ^33^, ^34^ ^]^ Consequently, the macrochain assemblies tend to adopt a 3D architecture. This structural transformation is accompanied by a rise in viscosity and, leveraging the reinforcing attributes inherent to cellulose derivatives, a substantial boost in the hydrogel's resilience.^[^ ^33^, ^34^ ^]^ Therefore, the moderately HACC content ratio optimizes these competing factors to yield a hydrogel with the desired mechanical properties for effective application.

In the tensile testing of NACHG hydrogels, the moderately HACC content formulation manifested the highest maximum stress capacity, reaching 26.3 kPa. This significantly surpassed the tensile strengths observed in both the lowest HACC content and highest HACC content formulations (Figure 3D,E; Figure S1B, Supporting Information). The inferior tensile characteristics exhibited by hydrogels with a lower HACC ratio can be attributed to an excessively diluted state, predisposing them to dissolution and thus undermining their tensile resilience. Conversely, when the HACC concentration is elevated, the hydrogel's morphology deviates toward an amyloidal or granular consistency. Due to the diminished degree of cross‐linking between PNIPAM and HACC, coupled with the abundance of inter‐HACC cross‐linkages, the structural characteristics of the resultant composite material are significantly altered, resulting in a decrease in overall tensile performance. In the SEM analyses conducted at varied weight ratios of PNIPAM and HACC, a conspicuous phenomenon emerges as the proportion of HACC escalates, the resultant cross‐linked matrix manifests the presence of substantial agglomerations. This observation serves as compelling evidence in support of the heightened aggregation propensity of HACC within the composite structure. Turning to the assessment of porosity, a discernible trend is unveiled wherein systems with the minimal HACC content exhibit the most pronounced porosity (Figure S2B,C, Supporting Information). Porosity in the hydrogel reduces the effective bearing area, and as porosity increases, the mechanical strength decreases. The higher the porosity, the looser the structure of the hydrogel, leading to a diminished ability to resist external forces. In the group with a low proportion of HACC, large pores can lead to stress concentration, making material fracture more likely. For the group with a higher proportion of HACC, increased cross‐linking results in uneven pore distribution, which concentrates local stress and reduces mechanical strength. For drug release in the oral cavity, hydrogel structures with relatively large voids should be selected to facilitate drug release.^[^ ^35^, ^36^, ^37^ ^]^ Nonetheless, this enhancement in pore volume is accompanied by a compromise in the mechanical strength of the fabricated materials, alluding to a trade‐off between porosity and mechanical resilience.

In vitro fixation performance assessments of hydrogels on rat oral mucosal models provide a viable means to emulate the actual functional behavior of these hydrogels under wet adhesion conditions. The employment of shear tests in multiple orientations serves to replicate the intricate and dynamic movements encountered within the oral cavity during routine physiological activities. Specifically, vertical and horizontal shear forces were selected in this study to encapsulate the broad spectrum of oral movements (Figure 3F–I; Figure S1C,D, Supporting Information). Notably, the hydrogel group integrated with MN suction cup demonstrated significantly superior bonding efficacy compared to both the optimally formulated group devoid of suction cup (NACH2G0.2) and the control group. Upon incremental imposition of stress, a distinct transition in failure mode is observed at the critical point of delamination, indicative of a threshold beyond which adhesive integrity is compromised. The vertical shear strength exhibited by the MN suction cup‐integrated group surpassed that of the other groups by a factor of three, while horizontally, this enhancement was nearly quadrupled. This unique characteristic of the MN suction cup group, revealing a stepwise decrease in bonding strength concomitant with the detachment of individual suction cup, contrasts with the dissociation patterns observed in the NACH2G0.2 group and the control group, which exhibited varied patterns of chemical bond disruption.^[^ ^38^ ^]^ Consequently, the MN suction cup‐embedded hydrogel group emerged as the paramount adhesive, significantly outperforming its counterparts. This heightened adhesion capability underscores the exceptional performance and prospective utility of MN suction cup technology in wet adhesion applications, highlighting its potential as an innovative biomaterial with enhanced functional integration within moist biological environments.

### 2.4 NO Release Detection

In the context of drug release modulation, the thermoresponsive PNIPAM hydrogel facilitates the expulsion of encapsulated therapeutic agents when its lower critical solution temperature (LCST) of 32 °C is exceeded. This LCST serves as a crucial thermodynamic threshold beyond which PNIPAM undergoes significant conformational changes. Wound sites exhibiting inflammation typically maintain elevated temperatures, often reaching up to 37 °C, which amplifies the contraction behavior of PNIPAM.^[^ ^39^ ^]^ Upon surpassing the LCST, PNIPAM experiences a marked decrease in affinity for water molecules within its cross‐linked matrix, leading to the formation of intramolecular hydrogen bonds. This process induces the development of secondary structures, notably β‐sheet folding, which enhances the mechanical strength of the MN assembly and results in the spontaneous contraction of the hydrogel matrix. Consequently, this dynamic mechanism expels the encapsulated NO donor, GSNO, from the hydrogel network, thereby enabling an active mode of drug release. This sophisticated structural network thereby promotes proactive drug release, contributing significantly to the therapeutic facilitation of ulcerative wound healing. At a temperature of 25 °C, the NO release profile exhibited a comparable trend to that observed at physiological temperature (37 °C), attaining a maximum concentration of 18.1 µm after 4 h of incubation (Figure 3J). Notably, this peak concentration was inferior to the levels recorded at 37 °C, indicative of an enhanced NO discharge under elevated thermal conditions. Such findings suggest a promotional effect of higher temperatures on NO release, a phenomenon potentially beneficial for the restoration and healing of ulcerated surfaces compromised by infection. At a concentration of 500 µm (NACH2G0.2), the release of NO surpassed that observed at a lower concentration of 100 µm (NACH2G0.04). Specifically, the pinnacle NO release attained with 100 µm was recorded at 4.6 µm, a value beneath the threshold deemed physiologically efficacious. Consequently, the impact on ulcer remediation was marginal, implying inadequate stimulation of the healing process at this concentration (Figure 3K).

Ulcerative conditions associated with BIS are often accompanied by severe wound infections, which are exacerbated by anaerobic microbial metabolism, leading to a reduction in wound pH. Therefore, a comparative analysis of NO release at physiological pH (7.4) versus acidic pH (6.2) was carried out.^[^ ^40^ ^]^ The result showed that acidic environment facilitates NO release from GSNO, as evidenced by a pronounced elevation in NO release at pH 6.2, peaking within the initial 4‐h period. This increase, amounting to nearly 2.5 times the release observed at neutral pH, reaches a concentration of 22.4 µm, ensuring a robust initial drug delivery phase (Figure 3L). Considering physiological serum concentrations, NO is present within a nominal range not exceeding 60.0 µm.^[^ ^41^, ^42^ ^]^ The peak NO release attained from the studied system, being sub‐maximal in comparison to this natural upper limit, precludes the likelihood of cytotoxic NO overload, thereby ensuring therapeutic safety. Subsequently, the release rate transitions into a sustained, slower‐release profile conducive to prolonged therapeutic intervention, consistently exceeding the release rate observed at pH 7.4. In contrast, NO discharge at pH 7.4 shows a marginal increase initially, stabilizing after 4 h into a more gradual, sustained release pattern. This study demonstrates that, in the context of infected wounds, the hydrogel matrix incorporating multi‐nanolayered structures of the MN exhibits a dual‐release mechanism. Initially, a burst release phase is governed primarily by the thermoresponsive behavior of PNIPAM, triggered by temperature modulation and pH fluctuations. This is followed by a prolonged, sustained release phase facilitated by the inherent permeability characteristics of the hydrogel.

### 2.5 Antibacterial Properties

BIS, a condition predominantly precipitated by excessive bacterial proliferation culminating in pseudomembrane formation and severe infection, necessitates that the mitigation of bacterial contamination assumes paramount importance in clinical management strategies.^[^ ^43^ ^]^ Consequently, the integration of antimicrobial properties within hydrogel formulations becomes vital for the effective remediation of infected ulcerative surfaces. This investigation assessed the antibacterial efficacy of six distinct hydrogel formulations, differing in their composition ratios of MN constituents, alongside a HACC‐free group (NACG0.2), a PNIPAM‐free group (ACH2G0.2), a GSNO‐free group (NACH2), the oral ulcer gel OUG group, and a control group, with respect to their inhibitory action against *S. aureus* and *E. coli*.

**Figure 4:**
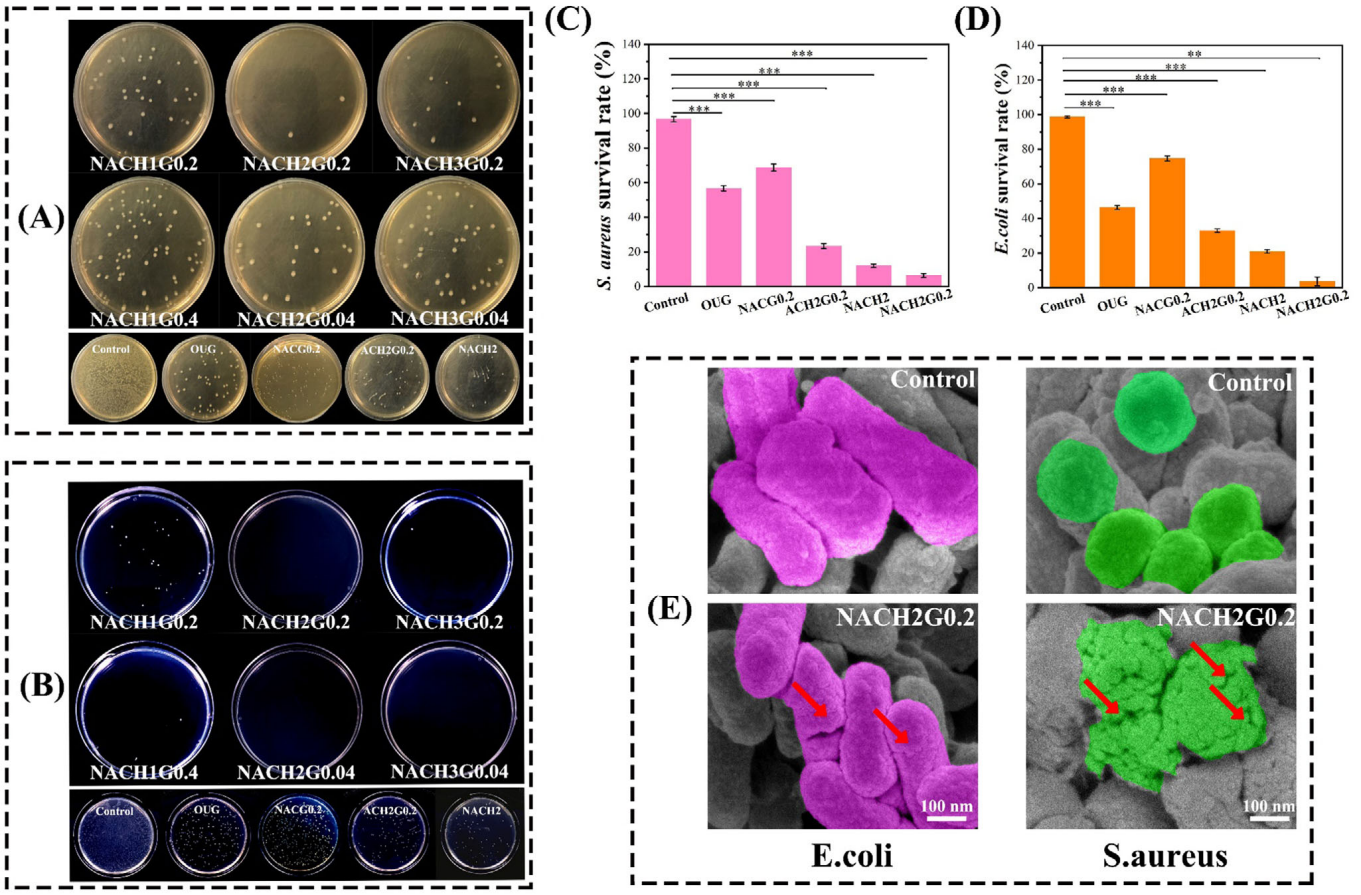
Antibacterial test of the synthesized materials. A) Photographs of MN hydrogels with various ratios against *S. aureus*. B. Photographs of MN hydrogels with various ratios against *E. coli*. C) Colony survival rates of the optimized group against *S. aureus* compared to each control group. D) Colony survival rates of the optimized group against *E. coli* compared to each control group (*n* = 3; **P < 0.05; ***P < 0.001). E) SEM image of *E. coli* and *S. aureus* (scale bar = 100 nm).

In the *S. aureus* antibacterial assays, the moderately HACC ratio composition group demonstrated superior inhibition rates compared to its counterparts (**Figure** **4**A), indicative of enhanced bactericidal potency potentially attributable to elevated GSNO concentrations. This heightened efficacy aligns with prior research suggesting GSNO's role in altering bacterial membrane electrostatic properties, thereby inducing cellular lysis.^[^ ^44^ ^]^ A similar trend was observed in the *E. coli* inhibition tests, where the moderately HACC ratio formulation again outperformed other groups in terms of inhibition efficiency (Figure 4B). Collectively, these findings underscore the moderately HACC ratio of MN components, in conjunction with high GSNO loading, as the optimal formulation for exerting maximum antibacterial activity. This formulation thus emerges as a promising candidate for advancing the treatment of infected oral ulcers, offering both targeted antimicrobial action and potential facilitation of ulcer surface healing.

Following the optimization process, the resultant MN ensemble was subjected to antimicrobial assays against *S. aureus* and *E. coli*, employing a control group for comparative evaluation of the bacteriostatic efficacy of the fabricated MN. The blank control group exhibited negligible bactericidal activity with *S. aureus* and *E. coli* (Figure 4C,D). The conspicuous reduction in inhibitory capacity observed in the NACG0.2 group can be attributed to the absence of the sterilizing efficacy conferred by HACC. It is noteworthy that HACC exerts its antimicrobial action through modulation of K^+^ and Ca^2+^ ion channel activities, thereby altering membrane permeability and inducing bacterial autophagy, as reported in references.^[^ ^45^, ^46^ ^]^ The ACH2G0.2 group lacked the characteristic drug release mechanism facilitated by the thermosensitive contraction of PNIPAM, while the NACH2 group failed to exploit the capability of GSNO in altering the electrostatic properties of bacterial cell membranes. Consequently, both groups demonstrated inferior bacterial inhibition performance compared to the NACH2G0.2 group. It was verified from SEM images of *E. coli* and *S. aureus* that GSNO and HACC can alter cell membrane permeability and lead to bacterial death through membrane cleavage (Figure 4E). Notably, when juxtaposed with the OUG hydrogel's bactericidal effectiveness, which stood at 43.3% and 53.7% for *S. aureus* and *E. coli*, respectively, the NACH2G0.2 MN patch group illustrated pronounced antimicrobial potency. The bactericidal capability was dramatically enhanced, achieving a complete eradication rate of 93.7% and 96.3% across all MN patches tested. This compelling evidence underscores the exceptional antibacterial characteristics of the fabricated MN patches.

### 2.6 Biocompatibility and Anti‐Inflammatory Assay

**Figure 5:**
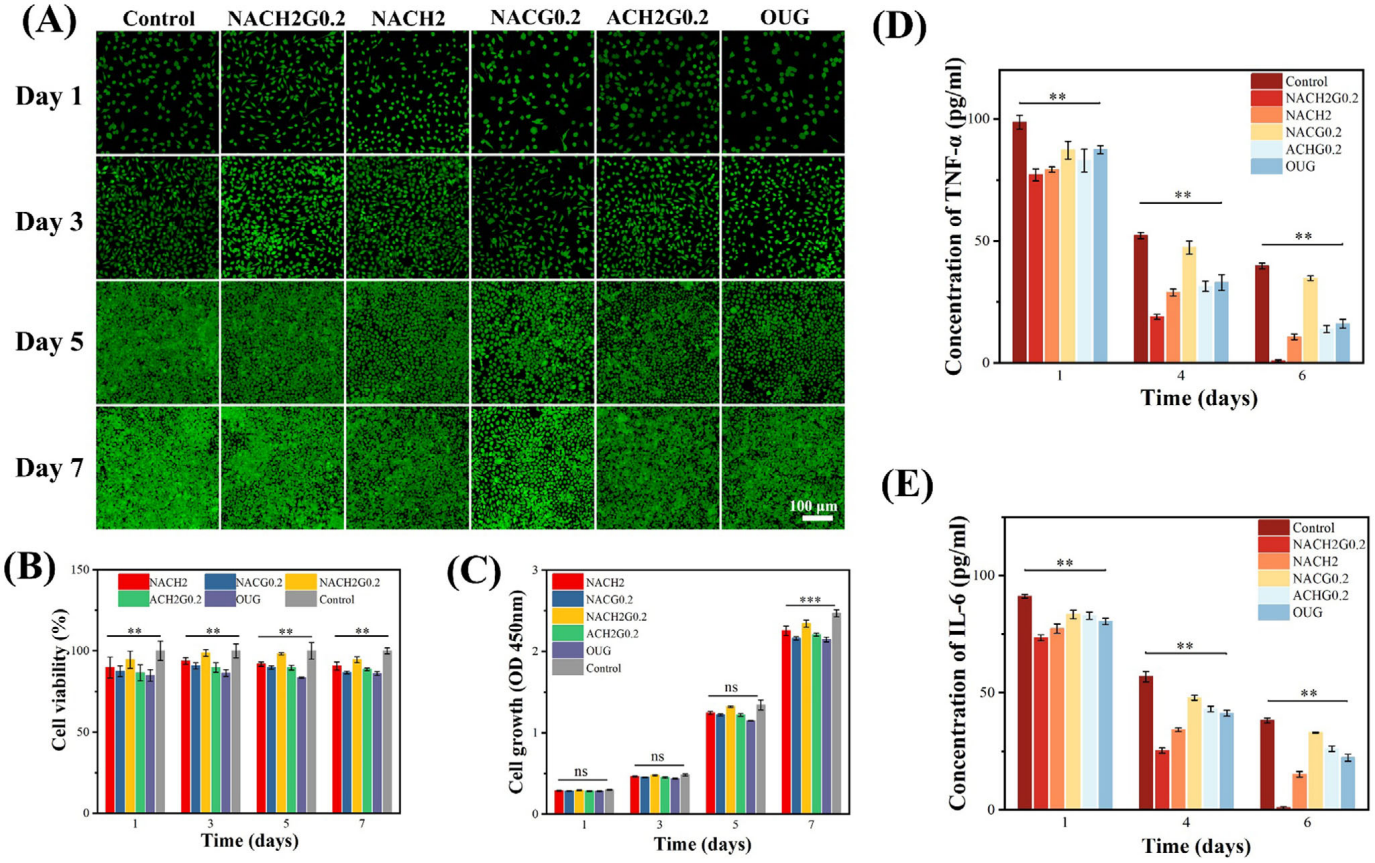
Biocompatibility of the MN patches. A) Live/dead cell staining images of L929 cells cultured with different samples after 1, 3, 5, and 7 days (scale bar = 100 µm). B) MTT assay for cell survival at 1, 3, 5, and 7 days for each group. C) MTT assay for cell growth rate of each group at 1, 3, 5, and 7 days. D) ELISA assay for TNF‐α concentrations on days 1, 4, and 6. E) ELISA assay for IL‐6 concentrations on days 1, 4, and 6. (*n* = 3 per group; **P < 0.05; ***P < 0.001; ns, not significant).

Despite the remarkable antimicrobial attributes of NACHG hydrogels, concerns have arisen over the potential cytotoxicity associated with elevated PAA concentrations, as documented in the literature.^[^ ^47^ ^]^ Consequently, an assessment of the cytotoxic profile of these hydrogels was conducted utilizing the 3‐[4, 5‐Dimethylthiazol‐2‐yl]‐2, 5‐diphenyltetrazolium bromide (MTT) assay, complemented by live/dead cell staining procedures. Throughout the MTT incubation period, the viability of cells exposed to all hydrogel formulations exceeded 85%, apart from the oral ulcer gel (OUG) group, which marginally fell to 84%. Notably, the optimal formulation (NACH2G0.2) displayed the highest viability outcome (**Figure** **5**B,C), suggesting that these hydrogels exhibit a low degree of cytotoxicity. This finding affirms adherence to biosecurity dosage limits for PAA, thereby ensuring no adverse effects on human physiology.^[^ ^48^ ^]^ Consistent with the MTT findings, live/dead cell staining analyses revealed a predominant population of viable cells (stained green), further corroborating the high biocompatibility profile of these materials (Figure 5A). These collective results reinforce the notion that the developed hydrogels maintain a favorable balance between antimicrobial efficiency and biocompatibility.

TNF‐α and IL‐6 are commonly used as markers for assessing inflammatory responses in cellular systems. The concentrations of these inflammatory cytokines in mouse oral mucosal epithelial cells were quantified using enzyme‐linked immunosorbent assay (ELISA). By day 6, the levels of TNF‐α and IL‐6 in the NACH2G0.2 group decreased to 0.90 and 0.94 pg mL^−1^, respectively, indicating a near‐complete resolution of the inflammatory response. This reduction was significantly more pronounced compared to the groups treated with HACC or GSNO alone. Notably, the TNF‐α and IL‐6 levels in the NACH2G0.2 group were markedly lower than those in the finished hydrogel OUG group, which exhibited concentrations of 16.05 and 22.31 pg mL^−1^, respectively, on day 6. These findings demonstrate the superior therapeutic efficacy of the combined administration of HACC and GSNO (Figure 5D,E).

### 2.7 Wound Healing Experiment

**Figure 6:**
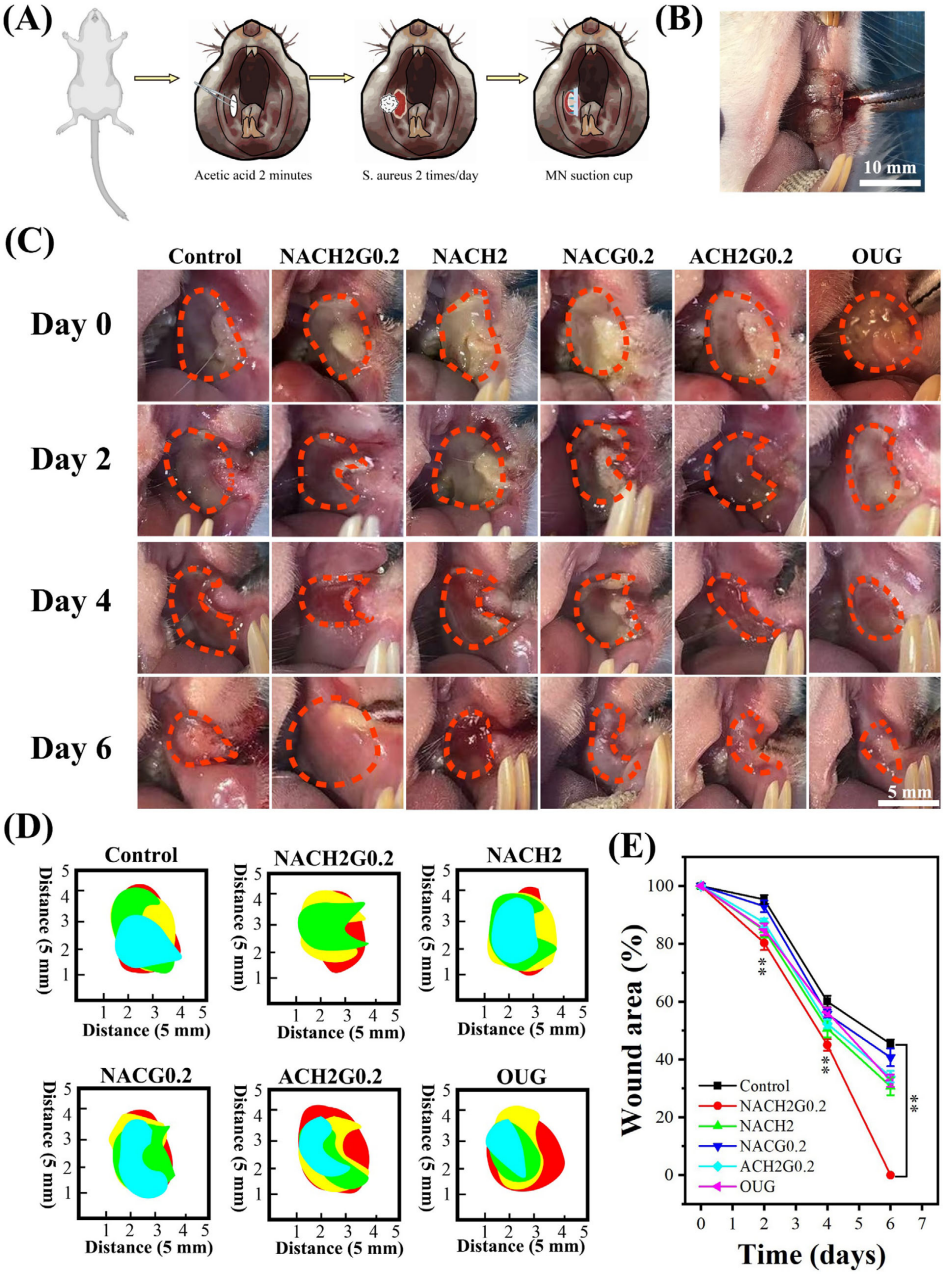
In vivo experiments. A) Procedure for establishing a rat model of BIS. B) Photographs showing the application of MN suction cup in the oral cavity of rats (scale bar = 10 mm). C) Wound healing progress with different MN treatments observed on days 0, 2, 4, and 6 (scale bar = 5 mm). D) Wound healing area comparison chart with different MN treatments observed on days 0, 2, 4, and 6. E) Wound healing trend analysis with different MN treatments observed on days 0, 2, 4, and 6 (*n* = 3 per group; **P < 0.05).

To assess the therapeutic efficacy of MN samples on infected ulcerations, a randomized trial was conducted involving rats inflicted with *S. aureus*‐induced BIS. All animal experiments were conducted in accordance with the requirements of the Medical Ethics Committee of Lanzhou University Stomatology School (No: LZUKQ‐2024‐041). These animals were organized into six distinct groups, each comprising three rats, as follows: i) Control group (Control), where infected ulcers received no intervention; ii) NO‐free group (NACH2), treated with GSNO‐absent MN patches; iii) Optimal group (NACH2G0.2), administered MN patches containing elevated GSNO concentrations tailored for enhanced therapeutic impact; iv) HACC‐free group (NACG0.2), where wounds were dressed with MN patches devoid of HACC; v) PNIPAM‐free group (ACH2G0.2), with wounds managed using MN patches lacking PNIPAM; vi) OUG group (OUG), with wounds treated using the commercial oral ulcer gel as a benchmark (**Figure** **6**A).

Sequential photographs of the ulcer sites were taken on days 0, 2, 4, and 6 to quantify the wound healing progression, considering the standard healing timeframe for such severely infected ulcers is approximately two weeks.^[^ ^49^ ^]^ The outcomes revealed that the NACH2G0.2 group demonstrated the utmost efficacy in expediting wound recovery, achieving complete ulcer closure by the sixth day. Conversely, in the Control group, while a reduction in wound dimensions and the thinning of the pseudomembrane were observed, these improvements were significantly less pronounced compared to the therapeutic interventions. In the GSNO‐free (NACH2) group, the pseudomembrane was entirely resolved, accompanied by epithelial regeneration. Notably, the residual unhealed ulcer area constituted approximately one‐half of that observed in the Control group. Conversely, within the NACG0.2 group, the pseudomembrane failed to efficaciously counteract infection, persistently covered by a non‐resolving pseudomembrane, exhibiting minimal divergence from the Control group's condition. The ACH2G0.2 group lacked the facilitation of ulcer surface healing through proactive mechanical contraction, resulting in incomplete ulcer recovery, with a disparity of roughly two‐fifths relative to the Control group concerning the healing extent. Evidently, the pseudomembranes in the OUG group showed negligible improvement in healing quality or reduction in size when juxtaposed against the Control group (Figure 6B,C). The outcomes of the animal studies convincingly demonstrated that the NACH2G0.2 MN patches expedited the healing process of infected ulcerative lesions in a markedly effective and swift manner. This group exhibited superior antimicrobial properties, concurrently achieving a substantial reduction in the healing duration required for BIS. The graphical representation of ulcer healing dynamics reveals that the NACH2G0.2‐treated group demonstrated a significantly accelerated recovery rate within a 6‐day timeframe. Evidently, a marked reduction in ulcerative area was observable as early as the fourth day of assessment. In juxtaposition to alternative control group, the NACH2G0.2 group accomplished full healing by the sixth day. An analytical depiction of the healing trajectory further underscores that while 32% of the original ulcerative area was not healed in the OUG group at the same juncture, underscoring the substantial therapeutic prowess and promise inherent to the NACH2G0.2 intervention strategy (Figure 6D,E).

### 2.8 Histochemical and Immunofluorescence Experiments

**Figure 7:**
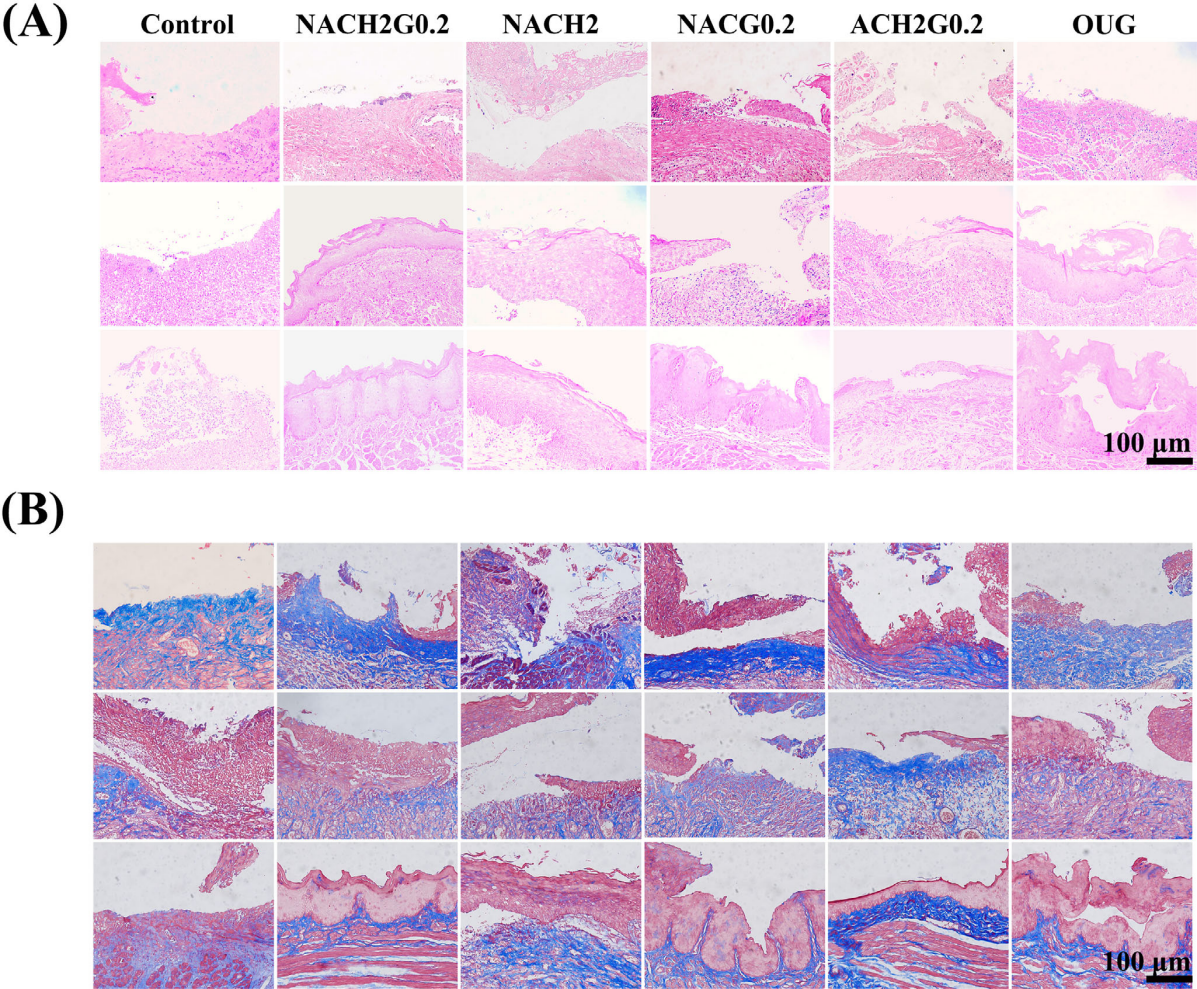
In vivo experiments. A) Representative images of the H&E staining on days 1, 4, and 6. B) Representative images of the Masson staining on days 1, 4, and 6 (*n* = 3; scale bars = 100 µm).

To meticulously evaluate the healing progression of ulcerated oral mucosa in rats with BIS, we employed Hematoxylin and Eosin (H&E) staining, Masson trichrome staining (Masson) alongside immunofluorescence (IF) techniques. Rodents from each group were selectively subjected to staining procedures on the 1st, 4th, and 6th day post‐treatment. Clear evidence of mucosal penetration by MN, discernible against the backdrop of normal tissue architecture, was noted (Figure S3, Supporting Information). On the initial day across all groups, bacterial infiltration and acute inflammatory responses were prevalent. The entire epithelial layer remained obscured, with the periphery of ulcers exhibiting irregular trauma. Epithelial cells at the ulcer base displayed aberrant gaps, and both the subepithelial region and ulcer peripheries manifested overt inflammatory signs, accompanied by vasodilation in select small blood vessels. By day 4, the control group exhibited a slight mitigation in inflammation and limited proliferation of stratum basale. In contrast, the NACG0.2, ACH2G0.2, NACH2, and OUG groups demonstrated a substantial decrease in inflammatory activity, coupled with the regeneration of both stratum basale and stratum spinosm. The NACH2G0.2 group, in particular, outperformed others, showcasing not only a pronounced attenuation in inflammation and cellular regeneration but also a conspicuous reduction in ulcer size (**Figure** **7**A). Progressing to day 6, residual inflammation, albeit diminished, persisted in the control group. Epithelial restoration progressed to the stratum spinosm; however, a considerable ulcerative area remained compared to the baseline assessment on day 1, highlighting the sustained healing challenge. In the NACG0.2, ACH2G0.2, NACH2, and OUG groups, the inflammatory response had largely abated, with a noticeable diminution in ulcerative areas compared to preceding observations. Notably, in the ACH2G0.2 and NACH2 groups, epithelial regeneration progressed to the stratum granulosum. Remarkably, within the NACH2G0.2 group, complete ulcer resolution was observed, accompanied by full restoration of the epithelial layer to the keratinized state (Figure 7A).

In Masson's trichrome staining, it is evident that the disruption of the epithelial cell layer leads to significant collagen deposition in the lamina propria and submucosa. This process is accompanied by a pronounced aggregation of fibroblasts, which facilitates the accumulation of types I and III collagen, thereby aiding in the restoration of the epithelial barrier. However, following an initial 24 h period post‐treatment, all groups exhibited disorganized and chaotic collagen fiber arrangement, attributed to the infiltration of inflammatory cells. By the fourth day of treatment, a notable decrease in collagen deposition was observed in the NACH2G0.2, NACH2, ACH2G0.2, and OUG groups, concomitant with the regeneration of some epithelial cells and a reduction in inflammatory cell infiltration, leading to a gradual reorganization of the collagen fibers. On the sixth day post‐treatment, as the epithelium in the NACH2G0.2 group approached complete healing, the collagen architecture within the lamina propria and submucosa demonstrated a well‐ordered pattern, closely aligned with the stratum basale, with papillary projections extending into the regenerating epithelial layer. In contrast, the control group still presented with residual blood clots on day 4 and only minimal regeneration of the stratum basale and stratum spinosm by day 6, underscoring the superior therapeutic potential of the NACH2G0.2 regimen (Figure 7B).

**Figure 8:**
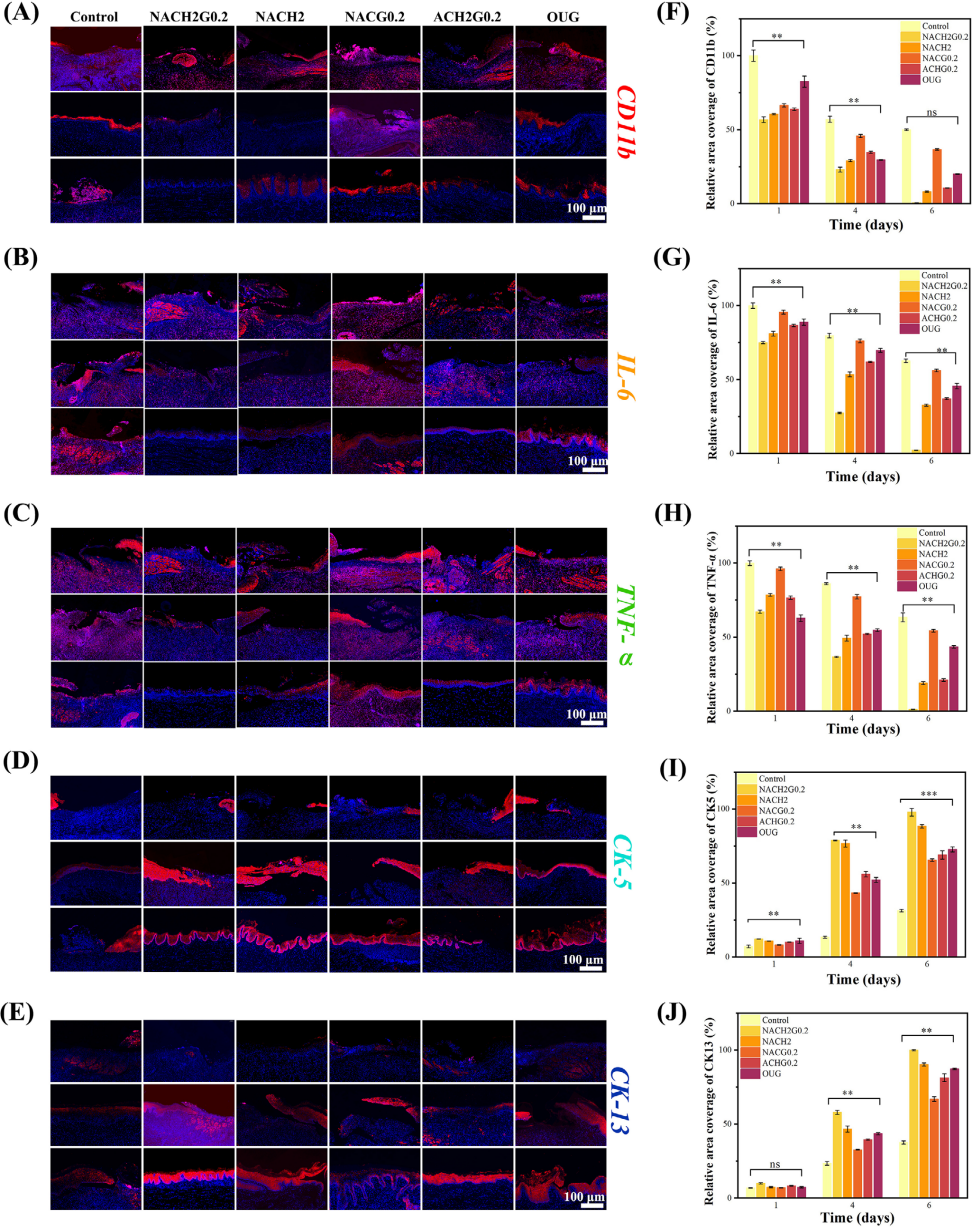
In vivo experiments. A) Representative images of CD11b on days 1, 4, and 6. B) Representative images of IL‐6 on days 1, 4, and 6. C) Representative images of TNF‐α on days 1, 4, and 6. D) Representative images of CK5 on days 1, 4, and 6. E) Representative images of CK13 on days 1, 4, and 6. F) Quantitative immunofluorescence analysis of CD11b. G) Quantitative immunofluorescence analysis of IL‐6. H) Quantitative immunofluorescence analysis of TNF‐α. I) Quantitative immunofluorescence analysis of CK5. J) Quantitative immunofluorescence analysis of CK13. (Scale bars = 100 µm; *n* = 3; **P < 0.05; ***P < 0.001; ns, not significant).

To assess the development of ulcerative epithelium and the associated inflammatory reaction, immunofluorescence methodology was employed utilizing five distinct markers: cytokeratin 5 (CK5), cytokeratin 13 (CK13), IL‐6, TNF‐α and cluster of differentiation 11b (CD11b).^[^ ^50^, ^51^, ^52^ ^]^ Specifically, the CK5 antibody was utilized to target the basal layer, thereby serving as an indicator for the proliferation of epithelial cells in this foundational stratum. Conversely, the CK13 antibody marked the intermediate and parabasal layers, providing insight into the progression of tissue repair and the healing rate. The IL‐6, TNF‐α, and CD11b antibody were instrumental in evaluating the inflammatory activity within the mucosal epithelium. Notably, across all experimental groups, conspicuous CD11b immunoreactivity was observed on the initial day (day 1), affirming the initiation of an inflammatory response. This response displayed a gradual diminution over time. By day 6, the control group retained noticeable CD11b staining, indicative of persistent inflammation. Conversely, in the NACH2G0.2 treatment group, a substantial reduction in CD11b staining was evident as early as day 4, suggesting a potent anti‐inflammatory efficacy associated with this intervention, further validating the superior anti‐inflammatory properties of the NACH2G0.2 group's therapeutic regimen (**Figure** **8**A). IL‐6 is a soluble small molecule pro‐inflammatory factor, which can be seen in the expression of immunofluorescence, and the expression of IL‐6 spreads throughout the whole layer of the tissues (Figure 8B). TNF‐α is mainly active on the cell surface in the form of transmembrane peptide, and immunofluorescence shows that the expression point is mainly concentrated in the pericellular area (Figure 8C). In the NACG0.2 group, the absence of an antimicrobial effect resulted in the weakest reduction of inflammation. In contrast, the NACH2G0.2 group showed significant improvement, with inflammation subsiding noticeably by day 4 and being almost completely resolved by day 6, owing to the dual anti‐inflammatory effects of HACC and NO. HACC exerts its anti‐inflammatory effects through its antimicrobial properties, thereby reducing the host's immune response. Simultaneously, GSNO promotes vasodilation, facilitates the recruitment of growth factors, and aids in the removal of metabolic byproducts, collectively exerting a dual modulatory effect.^[^ ^53^ ^]^ Consistent with the results of in vitro experiments, NO had a significant anti‐inflammatory effect. Consistent with the immunofluorescence intensity analysis, NACH2G0.2 showed optimal growth performance, and the intensity of TNF‐α, IL‐6 and CD11b had basically achieved complete disappearance on day 6, indicating that the inflammatory response had been basically eliminated completely. On day 6, the fluorescence coverage of TNF‐α in the NACH2 and NACG0.2 groups was 19% and 54.3%, respectively, and the fluorescence coverage of IL‐6 and CD11b followed the same trend as TNF‐α, suggesting that HACC and NO have certain anti‐inflammatory abilities, respectively, while the two superimposed can achieve optimal inflammation clearance (Figure 8F–H).

This synergistic interplay results in a more efficacious attenuation of the inflammatory response compared to other study groups, underscoring the superior therapeutic potential of this combinatorial approach. On the fourth day of the experiment, CK5 expression was discernible, with a noticeable presence of epithelial cells observed in the control group. However, this observation was markedly inferior in quantity compared to those in the NACG0.2, ACH2G0.2, NACH2, OUG, and NACH2G0.2 groups (Figure 8D). Notably, these comparative groups also exhibited pronounced immunoreactivity for CK13, in addition to CK5, highlighting a broader differentiation profile. Specifically, the NACH2G0.2 group demonstrated a statistically significant increase in the vertical growth of cells within the stratum spinosm when juxtaposed against all other experimental groups. On the sixth day, while the control group exhibited evident CK13 staining, the height of the cell layer remained considerably lower when contrasted with the remaining groups (Figure 8E), suggesting that while proliferation of the stratum spinosm had indeed initiated, their rate of growth was substantially slower. This disparity underscores the differential dynamics of cellular development across the various experimental conditions. In agreement with the outcomes derived from immunofluorescence intensity assessments, the NACH2G0.2 group exhibited superior growth characteristics. Notably, the CD11b immunoreactivity experienced a near‐complete attenuation by day 6, implying the effective resolution of the inflammatory response. Furthermore, the consistently elevated expression levels of cytokeratins CK5 and CK13 across control groups signified accelerated proliferation and favorable maturation of the epithelial cell population. Relative to the OUG group, NACH2G0.2 not only exerted a pronounced anti‐inflammatory action but also facilitated epithelial proliferation, thereby validating the therapeutic efficacy in the NACH2G0.2‐treated group (Figure 8I,J). Significant augmentation in the vertical dimension of the stratum spinosm was recorded in the NACG0.2, ACH2G0.2, OUG, and NACH2G0.2 groups, indicative of accelerated cellular proliferation. The remarkable performance of the NACH2 group can be primarily attributed to the bactericidal properties of HACC, which effectively curtails inflammation and thereby suppresses the upregulation of IL‐6, IL‐1β, and tumor necrosis factor‐β (TNF‐β).^[^ ^54^, ^55^ ^]^ In the ACH2G0.2 group, the elevated growth rate of stratum spinosm was strongly correlated with the antimicrobial and GSNO anti‐inflammatory capacities of HACC, but its active contractile capacity without PNIPAM made it second to the NACH2G0.2 group in terms of treatment.

Furthermore, the disintegration of GSNO leads to the release of NO, which facilitates vasodilation, thereby enhancing the accumulation of growth factors such as epidermal growth factor (EGF), transforming growth factor alpha (TGFα), fibroblast growth factor (FGF), among others, collectively stimulating epithelial proliferation.^[^ ^54^, ^55^ ^]^ The NACH2G0.2 group showcased comprehensive epithelial layer restoration with complete ulcer healing, underscoring the pivotal role of PNIPAM's dynamic mechanical contraction in facilitating the recovery of the ulcerated surface. This multifaceted approach, integrating anti‐inflammatory mechanisms, growth factor recruitment, and the unique physicochemical properties of the applied materials, collectively expedited the healing process and reinstated tissue integrity. Consistent with the immunofluorescence intensity analysis, NACH2G0.2 showed optimal growth performance, with CK5 and CK13 intensities consistently higher in all control groups, indicating faster and well‐developed epithelial cell growth. There was also a good anti‐inflammatory effect and promotion of epithelial growth compared to OUG, demonstrating the effectiveness of treatment in the NACH2G0.2 group.

## 3 Discussion

In recent years, significant strides have been made in the treatment of oral ulcers, particularly in the realm of integrating biomimetic strategies, which have unveiled new therapeutic pathways. The oral environment, characterized by the presence of saliva, creates a mucosal layer that impedes hydrogel adhesion and drug release. However, MN, as a localized drug delivery system, can penetrate both the mucosal layer and the mucous membrane surface to reach deeper tissue layers for drug release.^[^ ^56^, ^57^ ^]^ Our innovation, MN suction cup inspired by the tarsal claws of Drosophila, effectively adheres in moist environments.^[^ ^58^, ^59^ ^]^ Drawing inspiration from Zhu et al.’s^[^ ^38^ ^]^ bionic blue‐ringed octopus design, the suction cup concept aids wet environment adhesion. In this study, the suction cup only provided anchorage, but when combined with the MN design inspired by Drosophila tarsal claws, we achieved enhanced fixation and active drug delivery. PVA hydrogel was utilized for the suction cup, while the MN and substrate were fabricated from NACHG hydrogel. PVA's ability to form numerous hydroxyl groups (‐OH) upon polymerization enhances adhesion through hydrogen bonding with water molecules in the oral mucosal layer. These hydroxyl groups can also form strong covalent bonds with various compounds in the oral cavity. The suction cup's negative pressure environment, enhanced by atmospheric pressure, boosts adhesion performance in moist conditions. Additionally, the hydrogel in the MN component forms amide bonds and hydrogen bonds to achieve chemical bonding. Together, hydrogen bonding, chemical bonding, and atmospheric pressure contribute to exceptional adhesion in wet environments.

Many current hydrogels passively facilitate wound healing by delivering drugs and growth factors. In contrast, PNIPAM actively promotes healing through thermal contraction, releasing drugs while generating contractile forces.^[^ ^60^, ^61^ ^]^ Inspired by the embryonic wound closure process, Blacklow et al.^[^ ^62^ ^]^ ingeniously devised an active adhesive dressing (AAD) system. During embryonic healing, actin cords form at wound edges, generating contractile forces to close the wound. PNIPAM's mechanical contractile force has been affirmatively validated. Utilizing GSNO on this platform further promotes active ulcer healing, enhancing the rate of recovery. Ulcer surfaces are often infected with bacteria such as *S.aureus* and *E.coli*, which form a pseudomembrane. Quaternary ammonium salts disrupt ion metabolism and lyse cell membranes, effectively killing bacteria and preventing pseudomembrane formation.^[^ ^19^ ^]^ GSNO, upon contact with blood and other physiological environments, can break the N═O double bond, releasing NO and promoting ulcer healing.^[^ ^21^ ^]^ Since BIS is an infected ulcer surface, its temperature is higher than body temperature, enhancing PNIPAM contraction, which increases drug release for short‐term treatment and allows slow release over time.^[^ ^63^ ^]^ Additionally, bacterial acid production acidifies the ulcer microenvironment, accelerating NO release. Trials of MN suction cup patches demonstrated clinical healing in rat oral ulcers within 6 days. Unlike exogenous growth factor interventions, harnessing endogenous NO for healing aligns more closely with physiological processes, also it can effectively remove pro‐inflammatory factors such as TNF‐α and IL‐6, illuminating its significant potential in advancing ulcer healing therapies. This biomimetic approach to tissue regeneration highlights the compelling utility of NO in ulcer treatment.

MN suction cup show potential in oral soft tissue repair, improving both speed and extent of ulcer repair.^[^ ^64^ ^]^ However, frozen MN has limitations due to restricted usage conditions and transport difficulties.^[^ ^65^ ^]^ Given the mere 20 s operational time of frozen MN, their clinical application demands efficient handling and stringent storage conditions. Although studies have explored cell delivery with frozen MN, these constraints remain a considerable limitation. NO also serves as a vasodilator in soft tissue healing. In the context of ulcer healing, vasodilation enhances the delivery of oxygen, growth factors, and other essential nutrients while facilitating the removal of metabolic wastes produced by bacteria. Therefore, the mechanisms of vasodilation merit further investigation in the study of healing processes.

In conclusion, the MN suction cup patch technology offers dual advantages: it enhances local NO concentrations and demonstrates high biocompatibility and robust antimicrobial properties. Together, these features accelerate ulcer healing, underscoring the patches' significant potential in addressing infected ulcerative lesions and heralding promising advances in regenerative medicine and wound healing therapies.

## 4 Conclusion

In this study, we integrated bionic suction cup with MN to facilitate penetration into the mucosa, leveraging the suction cup to create negative pressure by expelling air. PNIPAM not only enables active drug release but also enhances healing through mechanical contraction applied to ulcer edges. Quaternary ammonium salts disrupt bacterial cell membranes, effectively eradicating pathogens. GSNO facilitates NO release, promoting vasodilation and subsequent ulcer healing. Tailored to the unique microenvironment of bacterial infectious stomatitis ulcers, the NACHG hydrogel MN targets the disease's underlying causes, accelerating the healing process. The MN suction cup engineered in this study exhibit multiple functionalities, including mechanical contraction, antimicrobial and anti‐inflammatory properties, and controlled NO release. They are versatile, suitable for treating various ulcers of differing sizes and locations, yielding superior and faster healing outcomes. This innovative approach holds promise for significantly improving therapeutic efficacy in the management of oral ulcers.

## 5 Experimental Section

### Materials

Acrylic acid (AA), hydroxypropyltrimethyl ammonium chloride chitosan (HACC), S‐nitrosoglutathione (GSNO), N‐isopropylacrylamide (NIPAM), N, N‐methylenebisacrylamide, ammonium persulfate, sodium carboxymethylcellulose, and poly (vinyl alcohol) (Mn: 31,000 to 50,000) were all purchased from Sigma‐Aldrich (USA). Jinlikang Oral Ulcer Gel (OUG) was obtained from Qinghai Qilikang Medical Devices Co. PDMS was sourced from Dow Chemical (USA). LB broth was acquired from Shanghai McLean Biochemistry Co. The MTT assay, live/dead cell double‐staining kit (Calcein‐AM/PI), Griess assay kit were provided by Dalian Meilun Biotechnology Co. Ltd, Mouse IL‐6 Uncoated ELISA kit (88‐7064, Thermo Fisher Scientific) and Mouse TNF‐α Uncoated ELISA kit (88‐7324, Thermo Fisher Scientific). H&E staining solution and the following antibodies were used: Anti‐Cytokeratin 13 antibody (CK13, Abcam), anti‐CK5 antibody (Bioss), TNF‐α monoclonal antibody, IL‐6 monoclonal antibody and CD11b monoclonal antibody (Thermo Fisher Scientific).

### Cell Culture

L929 cells (X120311, Shanghai Fusheng Industrial Co. Ltd, China) and Mouse Oral Mucosal Epithelial cells (ABC‐TC4366, ACCEGEN) were cultured in Dulbecco's modified Eagle's medium (Gibco) with 10% fetal bovine serum (Gibco) and 1% penicillin and streptomycin (100 IU ml^−1^) at 37 °C under 5% CO_2_.

### Experiment Animal

Male Sprague‐Daley rats (250 ± 10 g) were provided by the Animal Experiment Center of Lanzhou University. All animal experiments were conducted in accordance with the requirements of the Medical Ethics Committee of Lanzhou University School of Stomatology (No. LZUKQ‐2024‐041). All animals were fed standard laboratory diets, subjected to 12h/12h light/dark cycles under specific pathogen‐free conditions, and underwent environmental acclimatization for at least 1 week before any animal experiments.

### Material Synthesis

Quaternary amine chitosan in amounts of 50, 100, or 150 mg, along with 200 mg of PNIPAM, was added to 2 mL of water and ultrasonicated for 20 min. Separately, 50 mg of sodium carboxymethyl cellulose was added to 1 mL of water. Another solution was prepared by dissolving 5 mg of N, N‐methylenebisacrylamide and 30 mg of ammonium persulfate in 1 mL of water. These three solutions were then thoroughly mixed, followed by the addition of 100 µL of AA, and stirred with a magnetic stirrer for 2 h. Finally, GSNO at concentrations of 100 or 500 µm was added and stirred with a magnetic stirrer for 2 min to complete the NACHG hydrogel.

To prepare a PVA solution, 200 mg of PVA was weighed and added to 3 mL of distilled water. This mixture was heated to 90 °C with stirring, using a thermostatic water bath. Once the PVA was fully dissolved, the solution was maintained at 60 °C for 30 min to remove air bubbles and then rapidly cooled in a refrigerator set to −20 °C. After 14 h, it was removed and thawed at room temperature for 6 h.

### 3D Model Printing and Synthesis of MN Suction Cup

The design process utilized 3DS MAX 2024 software. Each MN was designed with a base radius of 250 µm and a height of 700 µm. The patch measured 1 cm × 1 cm and incorporated four suction cups, with MN positioned inside the suction cup. A light‐curing micro‐nano printer (S230, BMF Precision Technologies) was used to 3D print the resin model. An intermediate mold was employed to create a negative mold for PDMS preparation. The NACHG hydrogel was first cast to form the MN component, then PVA hydrogel was filled to the mold's top. The construct was placed in a −20 °C environment overnight and subsequently frozen in a −80 °C freezer for 4 h to form the MN suction cup.

### Hydrogel Morphology Observation and Chemical Structure Detection

The morphology of the optimal MN group was analyzed using scanning electron microscopy (SEM) (JSM‐6701F, JEOL, Japan). Fourier transform infrared spectroscopy (FTIR) (Nicolet iS50, Thermo Fisher Scientific, USA) was employed to record the IR spectra. X‐ray diffraction (XRD) spectra were obtained using a Rigaku D/Max‐2400 diffractometer. Images of gelatin post‐MN puncture were captured using a microscope (VERT1, Zeiss, Germany).

### Tensile Test

The tensile speed was set to 100 mm·min^−1^. Tensile stress was calculated as σ_b_ = F/wt, where (F) is the force applied, (w) is the initial width, and (t) is the thickness of the specimen. Tensile strain (ε_b_) is defined as the elongation relative to the initial scale length of the gel sample. The elastic modulus (E) is determined as the slope of the stress‐strain curve within the elastic region, i.e., E = σ_b_/ε_b_.

### Shear Test

The pure shear test was conducted using an Instron tester (Model 3342, with a pressure measuring element maximum of 50 N) by applying unidirectional tension to hydrogel samples measuring 80 mm × 5 mm × 1.5 mm. A volume of 200 µL of each hydrogel was applied to the surface of rat buccal mucosa, with another piece of skin tissue placed atop and gently pressed. The hydrogel‐coated mucosa was maintained at 37 °C for 3 h. Shear testing was carried out at a rate of 2 mm min^−1^ to evaluate the hydrogel's properties.

### Peel Adhesion Test

A standard 180° peel adhesion test was performed using the same Instron machine to evaluate adhesion performance. The adhesion energy was calculated as (2 × F/w), where (F) is the constant peeling force and (w) is the specimen width. Rat buccal mucosa was used for the test, with a peeling rate fixed at 100 mm min^−1^. After the application of 200 µL of hydrogels to the buccal mucosa and the pressing of a second skin tissue layer, the assembly was incubated at 37 °C for 3 h.

### Penetration Ability

To assess the puncture capability of the hydrogel MN, a magenta solution was mixed with 8 wt.% gelatin to simulate the mucus layer and oral mucosa. Images of the puncture process were captured at 0, 20, and 40 s.

### Compression Properties

The mechanical properties of the MN component were tested using a displacement force test rig (UTM2102, Shenzhen Sun Technology Co., Ltd., Shenzhen, China). The MN patch was mounted on a vertically positioned, rigid platform with the MN facing upward. The compression capacity was evaluated by moving the test bench sensor vertically toward the MN at a speed of 2 mm min^−1^.

### Characterization of Thermo‐Response

The thermo‐response was characterized by placing the samples in artificial saliva at 37 °C and measuring the volume change over time. The initial and final dimensions were noted as L_0_ and L, respectively, and the area strain was calculated as 1 – (L_0_/L)^2^.

### Solubility Assay

Hydrogels of similar size and shape from different groups were immersed in artificial saliva at 37 °C and removed at regular intervals. Excess water was blotted off using filter paper, and the hydrogels were weighed. The swelling ratio was calculated until the weight of the sample ceased increasing, using the formula (W_t_ – W_0_)/W_0_ × 100%, where (W_0_) is the original weight and (W_t_) is the weight at a specific time.

### In Vitro Degradation of Hydrogels

Lyophilized hydrogel samples (≈100 mg, *n* = 3) were immersed in 3 mL of artificial saliva at 37 °C. At specified intervals, the samples were removed, rinsed with deionized water to remove excess salt, dried, and weighed. The degradation rate (DR) was calculated as DR = (W_0_ – W_t_)/W_0_ × 100%, where (W_0_) is the initial weight, and (W_t_) is the weight at the designated time.

### Griess Experiment

A standard curve was developed based on the concentration of the standard solution, exhibiting linearity. Optical density (OD) values were recorded at pH 6.2 and 7.4 at 1, 2, 3, 4, 6, 12, and 24 h. NO release was plotted according to the standard curve, with similar measurements taken at temperatures of 25 and 37 °C.

### Antimicrobial Performance

In the experiments, the antimicrobial properties of MN were tested by plate colony counting method. First, *E. coli* (ATCC 33525, Warner Bio) and *S. aureus* (ATCC 25923, Shanghai Beinuo Biotechnology Co., Ltd.) were diluted to 1 × 10^−6^ mL^−1^ during the active growth period. Next, different concentrations of NACHG hydrogel (4: 2: 1: 1: 0.04, 4: 2: 1: 1: 0.2, 4: 2: 1: 2: 0.04, 4: 2: 1: 2: 0.2, 4: 2: 1: 3: 0.04, 4: 2: 1: 3: 0.2) and NACG0.2, ACH2G0.2, NACH2, and OUG were co‐cultivated with equal volumes of diluted bacterial solution for 24 h at 37 °C. Finally, the co‐culture solution was diluted again to 1 × 10^−6^ mL^−1^ and inoculated on 200 µL agar plates. photos were taken after incubation at 37 °C for 24 h, and the number of surviving bacteria was counted by plate counting.

### In Vitro Cytotoxicity Assay

The cytotoxicity of the MN hydrogels was evaluated using the MTT assay. L929 cells (X120311, Shanghai Fusheng Industrial Co. Ltd, China) were separated into five groups and incubated with equal‐quality hydrogels (Control, NACG0.2, ACH2G0.2, NACH2, and OUG) in 96‐well plates for four days. Each group had three parallels, with an initial concentration of 2 × 10^4^ cells mL^−1^ per well. Daily, the materials were transferred to new sterile wells, followed by the addition of medium with 10% MTT solution and incubation for 4 h. Post‐incubation, the medium was removed, and 400 µL of dimethyl sulfoxide was added to dissolve formaldehyde crystals. Cell viability was determined by measuring optical density (OD) values.

L929 cells were also co‐cultured with varying hydrogels for periodic assessments (1, 3, 5, and 7 days). Cell activity was observed using a live/dead cell double‐staining kit (Calcein‐AM/PI, 100 µL per well) at room temperature for 30 min, and observed under an inverted fluorescence microscope, with live cells appearing green and dead cells appearing red.

### In Vitro ELISA Assay

Each well of 24‐well plate was inoculated with 8 × 10^5^ mouse oral mucosal epithelial cells, which were induced using lipopolysaccharide (LPS) for 24 h and then co‐cultured by adding materials from each group. The cell supernatants were taken on days 1, 4, and 6 respectively to detect changes in the concentrations of TNF‐α and IL‐6 using ELISA kits.

### Animal Model Establishment

Eighteen 6‐week‐old male Sprague‐Dawley rats, weighing ≈250 to 300 g, were obtained from the Animal Experiment Center of Lanzhou University. The animal experimental procedures were approved by the Animal Ethics Committee of Lanzhou University School of Stomatology (No. LZUKQ‐2024‐041). To induce local mucosal trauma, a small 0.5 cm^2^ piece of paper soaked in 40% glacial acetic acid was applied to the buccal mucosa of each rat for 2 min. This was followed by rubbing 20 µL of *S. aureus* bacterial solution onto the mucosa. The mucosa was treated twice daily, enabling the establishment of a BIS model after 2 days. Rats were anesthetized using an intraperitoneal injection of 10% chloral hydrate 0.3 ml/100 g combined with 0.03 mg kg^−1^ fentanyl (w/v).

### In Vivo Animal Experiment

The established animal model was randomly and evenly divided into six groups, with one group serving as the untreated control group. The remaining five groups with BIS were treated respectively with NACG0.2 MN patch, ACH2G0.2 MN patch, NACH2 MN patch, NACH2G0.2 MN patch, and OUG applied to the ulcerated surfaces. The rats' wounds were examined and treated daily, with photographs and documentation taken on days 0, 2, 4, and 6. Rats were euthanized on days 1, 4, and 6, and oral ulcer tissues were collected and fixed in 4% paraformaldehyde buffer for 24 to 48 h. The tissue samples were then dehydrated, embedded in paraffin, and sectioned into 5 to 7 µm thin slices for histological and immunofluorescence analysis of CK13 (BH‐01S4583, BHBT), CK5 (NB100‐77727FR, Novus), IL‐6 (GB12117‐100, Sevicebio), TNF‐α (GB12188‐100, Sevicebio), and CD11b (MAB1124‐SP, Bio‐Techne).

### Data Analysis

All data were expressed as the mean ± standard deviation (SD) and analyzed using Origin Pro 2022 or SPSS Statistics 22. Comparisons between treatment groups and the control group were conducted using two‐factor analysis of variance (ANOVA), with statistical significance set at *P* < 0.05.

## Conflict of Interest

The authors declare no conflict of interest.

## Supporting information



Supporting Information

## Data Availability

The data that support the findings of this study are available from the corresponding author upon reasonable request.
