# Supplementary material for: Bionic Microneedle Patch Inspired by Drosophila Tarsal Paws Boosts Healing in Bacterial Infectious Stomatitis
Source: Adv Sci (Weinh). 2025 Jun 23;12(35):e00432. doi: 10.1002/advs.202500432 (PMC12462995; doi:10.1002/advs.202500432)
Supplement: Supplementary file 1 — Supporting Information [file ADVS-12-e00432-s001.docx]

**Supporting Information**

**Bionic Microneedle Patch Inspired by Drosophila Tarsal Paws Boosts Healing in Bacterial Infectious Stomatitis**

*Jiaqi Qin, Cewen Hu, Huajing Zeng, Xiaohong Ran, Jie Zhang*, Bin Liu*, Zengjie Fan**

**
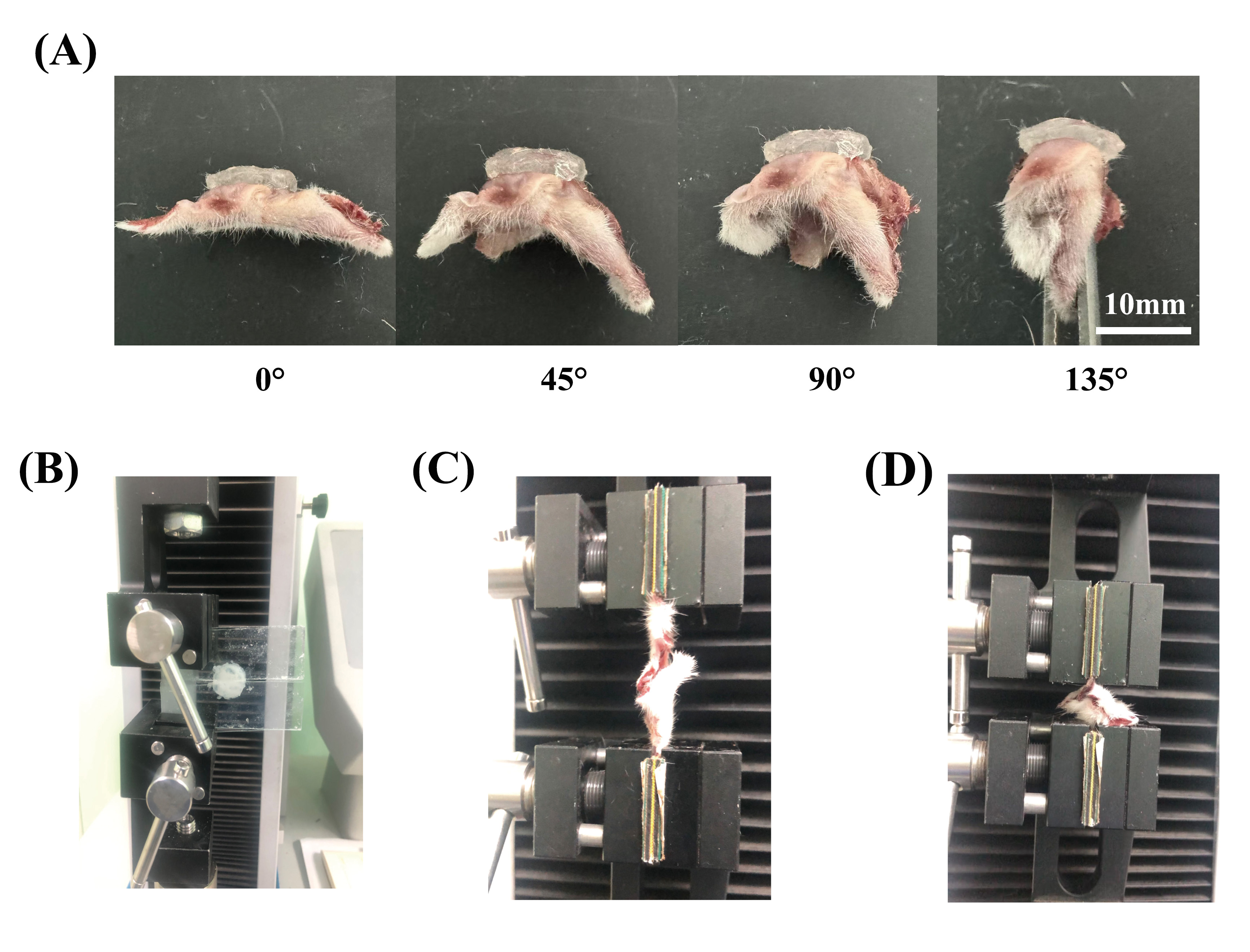
Figure S1**. A. MN suction cup bending adhesive properties with bending angles of 0°, 45°, 90°, 135° (Scale bar = 10 mm). B. Photographs of NACHG hydrogel in tension. C. Photographs of NACHG hydrogel in shear. D. Photographs of NACHG hydrogel in peeling.


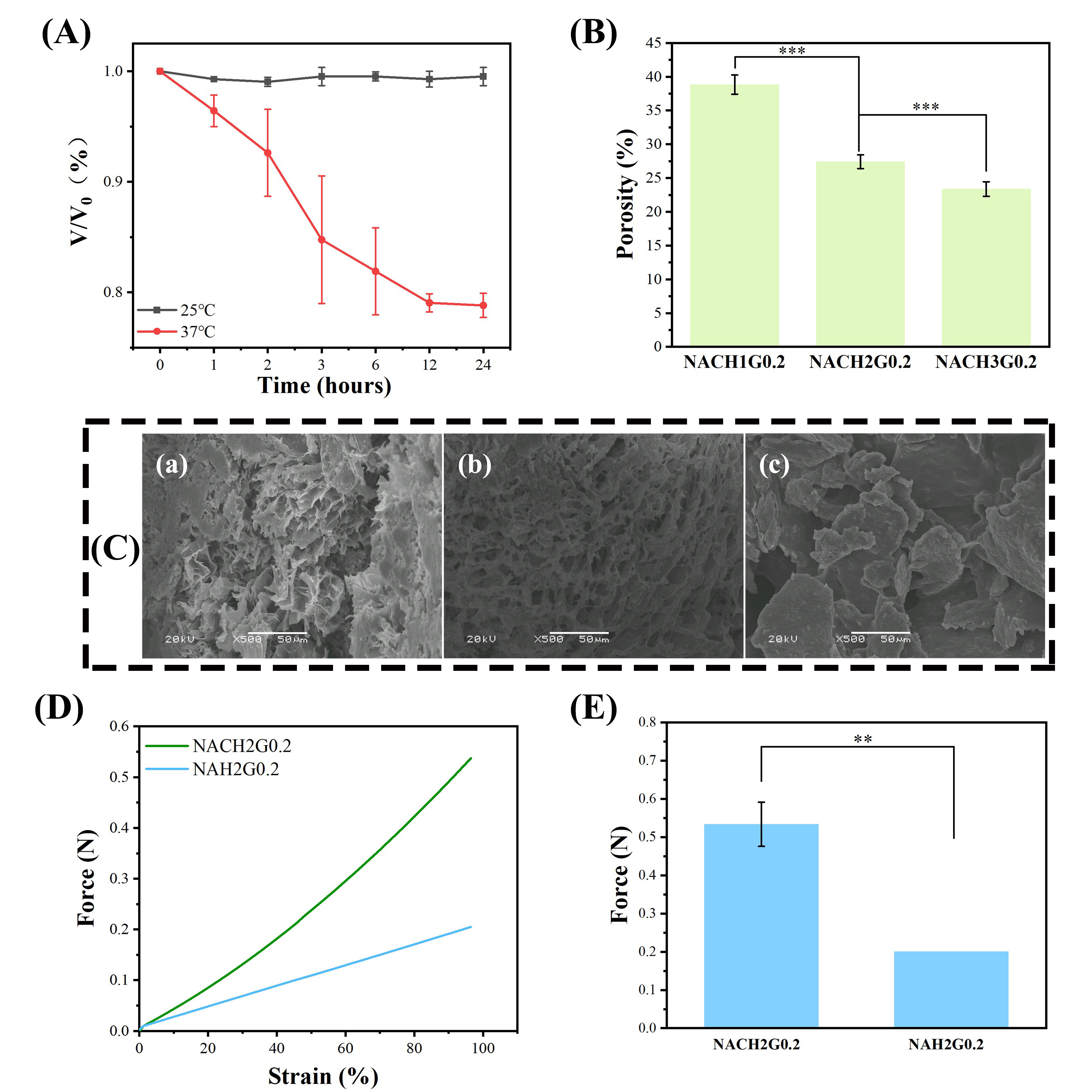


**Figure S2**. Characterizations of morphology and mechanical properties of MN suction cups. A. Proportion of thermal shrinkage of the NACH2G0.2 group of microneedle hydrogels within 24 hours at 25°C and 37°C. (n = 3). B. Comparison of porosity size at different PNIPAM and HACC ratios. (n = 3; ***P < 0.001). C. SEM images of MNs (scale bars = 50 μm). D and E. Compression properties of MNs in NACH2G0.2 and NAH2G0.2 groups without CMCNa (n = 3; **P < 0.05).


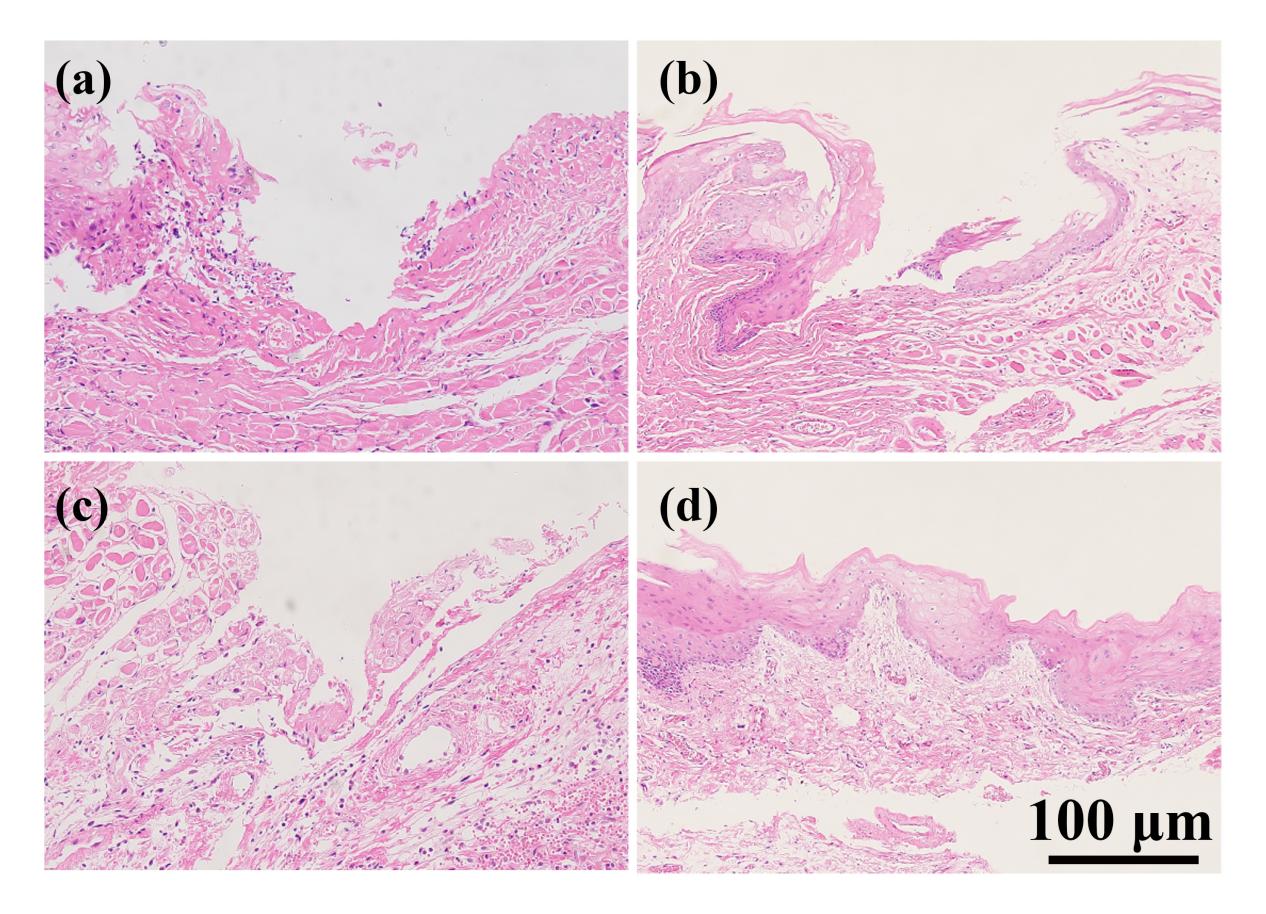


**Figure S3**. H&E staining of microneedle suction cup embedded in oral mucosa compared to normal mucosa. (a, b, & c) MN suction cup in oral mucosa; (d) normal mucosa (scale bar = 100 μm).

**Table 1**. Proportioning and naming of different groups.

| PNIPAM 200mg  PAA 100μL  CMC-Na 50mg | HACC 50mg | HACC 100mg | HACC 150mg |
| --- | --- | --- | --- |
| GSNO 100μM | NACH1G0.04 | NACH2G0.04 | NACH3G0.04 |
| GSNO 500μM | NACH1G0.2 | NACH2G0.2 | NACH3G0.2 |
